# Participatory ethnobotany: comparison between two quilombos in the Atlantic Forest, Ubatuba, São Paulo, Brazil

**DOI:** 10.7717/peerj.16231

**Published:** 2023-11-07

**Authors:** Thamara Sauini, Paulo Henrique Gonçalves Santos, Ulysses Paulino Albuquerque, Priscila Yazbek, Cremilda da Cruz, Eduardo Hortal Pereira Barretto, Maria Alice dos Santos, Maria Angélica Silva Gomes, Ginacil dos Santos, Silvestre Braga, Ricardo José Francischetti Garcia, Sumiko Honda, Priscila Matta, Sonia Aragaki, Anderson Ueno, Eliana Rodrigues

**Affiliations:** 1 Centro de Estudos Etnobotânicos e Etnofarmacológicos (CEE)—Departamento de Ciências Ambientais, Universidade Federal de São Paulo (UNIFESP), São Paulo, São Paulo, Brazil; 2 Laboratório de Ecologia e Evolução de Sistemas Socioecológicos (LEA), Centro de Biociências, Departamento de Botânica, Universidade Federal de Pernambuco, Brazil, Recife, Pernambuco, Brazil; 3 Associação dos Remanescentes de Quilombo do Cambury, Ubatuba, São Paulo, Brazil; 4 Herbário Municipal, Prefeitura do Municipio de São Paulo, São Paulo, São Paulo, Brazil; 5 Quilombo da Fazenda, Associação da Comunidade dos Remanescentes de Quilombo da Fazenda, Ubatuba, São Paulo, Brazil; 6 Centro de Estudos Ameríndios (CEstA), Universidade de São Paulo (USP), São Paulo, São Paulo, Brazil; 7 Herbário SP, Instituto de Pesquisas Ambientais (IPA), Secretaria de Meio Ambiente, Infraestrutura e Logística do Estado de São Paulo, São Paulo, São Paulo, Brazil

**Keywords:** Participatory research, Traditional communities, Quilombolas, Biodiversity, Conservation

## Abstract

Ethnobotanical studies that use the participatory research approach seek to involve the residents of a community in different stages of the study, promoting the registration, dissemination and strengthening of local knowledge, as well as the empowerment of decisions related to the sustainable use and management of resources. Using the participatory methodology, this study recorded and made a comparative analysis on the use of plants in two quilombola communities (Quilombo do Cambury-QC and Quilombo da Fazenda-QF) in the State of São Paulo. After a training on anthropological and botanical methods, local researchers selected and interviewed the local experts, recording their knowledge on plant uses and collecting the indicated plants, to be identified and deposited in herbariums. In addition, participant observation and field diaries were used by the academic researchers, helping to analyze the data. To test the differences in the composition of species known to local community, a Jaccard dissimilarity matrix was created, and a Permanova test was employed. During the 178 days of fieldwork, three local researchers from the QC and two from the QF, selected nine and eight experts on the uses of the plants in each quilombo, respectively, corresponding to 214 plant species, indicated for eight ethnobotanical categories. Our hypothesis has been confirmed, since the traditional knowledge found in both quilombos, regarding plant uses and the number of plant species by category, are distinct, since each community occupies particular plant areas and different phytophysiognomies. Most of the indicated species are native to the Atlantic forest, and no significant differences were observed in the proportion of native species *vs*. introduced among quilombos for any of the categories of use studied. Furthermore, the innovative methodology used, participatory ethnobotany, contributed to the empowerment of community members with regard to the use of their available resources in the environment in which they live, while retaining the intellectual property rights over their own knowledge.

## Introduction

Ethnobotany is the science that studies interactions between humans and plants in all their complexity, including knowledge, beliefs and cultural practices associated with the uses of these plants as foods, dyes, fibers, poisons, fertilizers, building materials for houses, oils, components of rituals, among others (Heinrich et al., 2004; Martin, 2004). For its applicability to be possible, many authors in Brazil, and in other countries have used indices that demonstrate the scope, versatility and conformity of the traditional uses of plants, both among members of the same community (Hanazaki et al., 2000; Amorozo, 2002; Canales et al., 2006; Albuquerque et al., 2007) and between different cultures (Prance, Balée & Boom, 1987; Begossi, 1996; Garibay-Orijel et al., 2007). In this approach, known as quantitative ethnobotany, the data analysis obtained offers knowledge about the most fragiles species, often because they have high versatility and importance of use, deserving greater attention in management plans, for example. Furthermore, they can indicate the need for sustainable forms of use, avoiding resource depletion.

Participatory research approach has been commonly applied (Goebel, 1998; Mosse, 2001; Johnson et al., 2004; Gilmore & Young, 2012), involving the active participation of members of a community, contributing to its empowerment in decisions about the use of available resources, and in promoting, among other aspects, the sharing of experiences with the community, seeking not only individual but also collective analyses. Basic local conservation is also a focus (Ericson, 2006; Sieber et al., 2014).

The knowledge of the residents of a community contributes to a better local environmental understanding, and their participation can occur in different ways, according to the type of research conducted and the local involvement obtained with the study. However, the methodological details developed in participatory research have been little explored. Grasser, Schunko & Vogl (2016) and Ericson (2006) discuss two forms of participation: active, where the community provides input about local problems and participates in decision-making; and passive, where community members are silent or donors of information, and the content included is controlled by people outside the community.

The different levels of involvement and participation of a community in a study can be observed in different works (Grasser, Schunko & Vogl, 2016; Cunha, Soares & Fraxe, 2011). Etkin & Ticktin (2005), for example, advocate participation in all stages of the research. The work carried out by Hitziger et al. (2016), between two Mayan peoples in Guatemala, is one of the few examples of published work where local communities participated with a high degree of involvement.

The works developed by our team using participatory ethnobotany methods, Rodrigues et al. (2020), Yazbek et al. (2019), and Sauini et al. (2020), were carried out in two quilombola communities, holders of a very diverse knowledge about the plant species of the Atlantic Forest in Brazil. In these works, participatory researches were carried out in conjunction with local researchers, who collected their plants and recorded their own knowledge. We define here participatory ethnobotany as a methodology involving the active participation of the community in the development of the research, since the definition of the study objectives, until the documentation of the knowledge itself, analyses and dissemination of data obtained. Still, members of the community are trained, from both botanical and anthropological perspectives, so that they can conduct the ethnobotanical survey themselves, with technical support by the academy. This method contributes to the empowerment of community members with regard to the use of their available resources in the environment in which they live, while retaining the intellectual property rights over their own knowledge.

Featuring approximately 20,000 species of plants, 8,000 of which are endemic, the Atlantic Forest is one of the terrestrial ecosystems with the greatest biodiversity and hosts several types of human communities that know and use the resources available (De Almeida et al., 2012;  Instituto Brasileiro de Geografia e Estatística (IBGE), 2018). Among these communities are the quilombolas, who live in areas called quilombos, and who developed a way of life linked to the land and the environment and are considered important holders of knowledge and practices related to the species found in their territories. Quilombola communities, which are characterized as ethnic-racial groups remaining from quilombo communities, have “its own historical trajectory, endowed with specific territorial relations, with a presumption of black ancestry related to resistance to the historical oppression suffered” (Brasil Constituição, 2003).

In this context, the present study focused on the survey and comparative analysis of plants used by residents of two quilombola communities, Quilombo do Cambury (QC) and Quilombo da Fazenda (QF), using the participatory ethnobotany method. We hypothesized that the traditional knowledge found in both quilombos, regarding the plant uses as well as the number of plant species per category, are distinct since they occupy particular areas and different phytophysiognomies.

## Methodology

### Ethical permissions

The following legal approvals were obtained for this research: (1) to access the Serra do Mar State Park area (COTEC n° 260108–009.510/2015); (2) to collect plants and access the Serra da Bocaina National Park (SIBIO n° 51199-1/2015 and n° 51199-2/2015); (3) to obtain prior informed consent and access traditional knowledge (SISGEN n° A648D14); (4) to be conducted by the Federal University of São Paulo (Research Ethics Committee n° 0452/2016 and n° 0843/2016); and (5) Quilombos’ Prior Consent Form (TAP): authorization from the representatives of the communities for the development of the study. UNIFESP’s Ethics Committee requires that researchers present their research registration in National System for the Management of Genetic Heritage (SISGEN). In order to carry out this registration, it is necessary that the research participants sign a document called Prior Consent Term, which authorizes access to their traditional knowledge through local leaders, in this case, the presidents of the associations of both quilombos.

### Human groups and study areas

**Figure 1:**
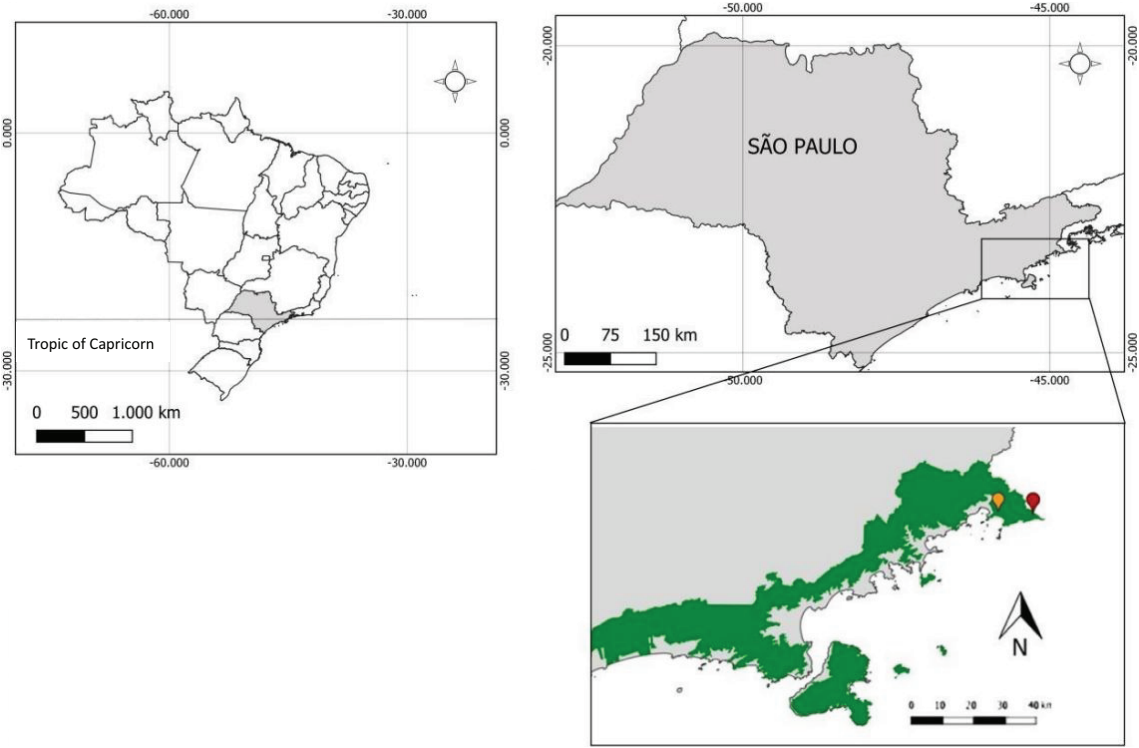
Location of Quilombo da Fazenda (in yellow), Quilombo do Cambury (in red) within the Serra do Mar State Park (in green), in Ubatuba, the state of São Paulo, Brazil.

The study was conducted in two quilombola communities: the Quilombo do Cambury (QC) and Quilombo da Fazenda (QF), located in the city of Ubatuba, in the State of São Paulo, Serra do Mar State Park (PESM)-Núcleo Picinguaba (Fig. 1). With an area of 332,000 hectares is the largest conservation unit (UC) in the Atlantic Forest and an extremely important region, because it is also the largest biological corridor in Brazil (PESM, 2018). Nevertheless, a portion of the QC is within the Serra da Bocaina National Park (PNSB), also located within the Serra do Mar, on the border between the states of Rio de Janeiro & São Paulo. With an area of 104,000 hectares and established in 1972, the PNSB shelters in its territory ecological refuges for endangered species, in addition to being an important component of historical and cultural heritage in the regions of Paraty and Minas Gerais (Instituto Chico Mendes de Conservação da Biodiversidade (ICMBio), Brasil, 2018).

The map (Fig. 1) was prepared by one of the authors (Sauini, T) using the free software QGIS, and a collection of spatial data from the National Institute of Colonization and Agrarian Reform and the Brazilian Institute of Geography and Statistics, and using the geographic coordinates reference system “sirgas 200” (Geocentric Reference System for the Americas).

According to the São Paulo State Forest Inventory (Inventário Florestal do Estado de São Paulo, 2020) and IBGE (Instituto Brasileiro de Geografia e Estatística (IBGE), 2012), both quilombos are located in the Atlantic Forest domain in phytophysiognomies of the Dense Rainforest. The QC is composed, in large part, of the Dense Submontane Rainforest and the Dense Montane Rainforest (the latter located in the northern portion of the quilombo), with a small stretch of Dense Rainforest of the Lowlands in the southwest. The QF is also composed of the Dense Sub Montane Rainforest and Dense Rainforest of the Lowlands, although with a greater presence of the latter vegetation. In addition, the QF includes a significant component of pioneer formation with fluvial influence, vegetation associated with alluvial plains, also called floodplains or swampy areas.

The formation of these communities followed the period of slavery, beginning in 1860, in the county of Ubatuba, when enslaved Blacks and ex-slaves fled from regions such as Ubatuba and Paraty and hid or simply occupied farmlands abandoned by bankrupt owners. Due to the geographic location of the two quilombos, the area remained isolated until 1970 when the BR 101 highway was built, thus favouring the movement of local residents and tourists and even the installation of electricity (FUNDART, 2014). It was defined as a Historical-Cultural-Anthropological zone, where the use of natural resources by these communities is allowed provided that there is resource management, thus supporting and strengthening the local way of life (São Paulo, 2006).

### Quilombo da Fazenda

The area of this quilombo was an old monoculture farm, initially of sugar cane and later of coffee, that depend on the labour of the enslaved Blacks and was called Fazenda Picinguaba. It was formed by Italian immigrants and descendants of African slaves who migrated to Brazil during the colonial period to work in the mill. There are approximately 170 residents in an area of 5,208.47 hectares, located in the opposite direction from the beach and characterized as a region of thick forest between the slopes of the Serra do Mar and the sea shore (Setti, 1985; Fundação Cultural Palmares, 2015).

The QF does not have a school or health center, and the nearest hospital is located 35 km away (Yazbek et al., 2019). The local religions are Roman Catholicism and evangelical Christianity, and the main source of income comes from the sale of handicrafts. Some families maintain their subsistence gardens, but many residents work in the city as waiters, painters, bricklayers and in other professions. Tourism is also a source of income for many residents who work as Park guides and environmental monitors.

The local food base consists of ingredients produced in the fields (such as manioc, yams and bananas) and vegetable gardens, as well as derivatives such as manioc flour and manioc bread (*beiju*). With easy access to roads, many of the ingredients that were previously produced in local gardens, such as coffee and sugar from cane, are currently purchased in city markets. There has also been the introduction of other industrialized foods such as crackers, meat, rice, butter, milk and cheese.

### Quilombo do Cambury

The QC was formed by enslaved Blacks and ex-slaves who were strategically hiding in this territory, and were difficult to access at the time. Today, it features two access routes, the main road to BR 101 and a small trail used by residents, which connects the road to the beach.

Because it is located on the coast, the QC is composed of a mixture of indigenous, Afro-Brazilian and European peoples, who gave rise to the Quilombolas and Caiçaras (fishermen) of the community. It includes approximately 300 people distributed among 50 families who live in a rich and diverse environment but face daily difficulties such as the lack of transportation, health care and education; and also the intense flow of tourists, which directly impact the region (ITESP, 2002, 2018). In addition, the community has a basic early childhood education school, two evangelical churches and a health post for basic health care, with the nearest hospital located 25 km from the community.

Food is produced through traditional, subsistence agriculture, and includes manioc for flour, sweet potatoes, beans, fruits, vegetables and others, grown in gardens in the backyards of the houses. Since the quilombo is located in a coastal region, its inhabitants also rely on fish, which are obtained from traps placed on Cambury beach, known as “enclosures”. Handicrafts and tourism, attracted by beautiful coastal scenery and many local campsites and restaurants, are the main sources of income for the community.

### Participatory ethnobotany

During a workshop held in 2015 at the Serra do Mar State Park—Núcleo Picinguaba, the managers of this Conservation Unit (CU) brought demands to the researchers of the universities present. One of them was the promotion of alternatives reconciling income generation and extraction of local natural resources legally by the residents of that CU.

Thus, the coordination of this project initiated meetings with the leaders of the two communities that occupy this area, in order to know how they could assist in local income generation and development from the plants used by communities. At first, the two main leaders were contacted (Mr. Zé Pedro, 11/30/1938-18/05/2021 and Mr. Genésio, 27/03/1927-12/01/2019), and they were responsible for forwarding the matter to the representatives of the Residents’ Associations. After several meetings between the project team (involving representatives of the academy and the community), it was concluded the need to register their knowledge before defining how these plants could be used for local development. Also, it was decided that some of the residents would participate in all phases of the study, from the design and documentation of local knowledge, training in anthropological and botanical methods, to the analysis and publication of data, to be accomplished through booklets and audiovisuals containing information about their practices: they are called here local researchers.

Approximately 1 year later, in May 2016, after all the authorizations were obtained, the fieldwork began, and it lasted until March 2018, totaling 178 days of fieldwork. Five quilombolas performed as local researchers: three women aged 39,46 and 50 years, in the QC (co-authors MASG, MAS and CC, respectively) and two men aged 35 and 42 years, in QF (co-authors GS and SB, respectively). Two of the QC researchers had been born and raised in the quilombo area, and the other was born in the city of Ubatuba, SP. They are artisans, cooks, and owners of a campground located within the quilombo. In the QF, one of the researchers is a descendant of enslaved Blacks in the region. When he was 18, married a local quilombola and started living in the community. The other was born and raised there. Both are artisans and experts on local plants and work in their home gardens and develop other activities when necessary.

In order to conduct the fieldwork, the academic researchers offered two trainings to the five local researchers: (1) anthropological methods, about how to conduct interviews with local experts (Bernard, 1988), and (2) botanical methods, for the collection of plants.

The local researchers also selected the interviewees of this study, here called local experts. The first local experts selected were the older individuals of the respective communities. Then, interviewees were selected according to their specialty. During fieldwork, it has been observed that certain genres are involved in specific knowledge—for example, experts in boat construction are usually men, not having been observed woman in this expertise, probably because it is an activity that involves a lot of physical strength and/ or due to local cultural issues. On the other hand, one of the men recognized as an expert in canoe construction also presented knowledge about food plants. This knowledge usually belongs to the women “known as cooks” in this same community. The knowledge about making crafts is widespread among men and women. Therefore, the influence of genders on the categories of plant uses was not observed, excepting for “shipbuilding” category.

Altogether, 17 local experts were interviewed adding the two quilombos: there were a total of nine in the QC, seven men and two women aged between 35 and 50 years old; and eight in the QF, five women and three men aged 43 to 81 years old. Five QC local experts had been born there and grew up and still live there. The others were born in the surrounding cities: Ubatuba, SP or Paraty, RJ. In the QF, one local expert was born in the quilombo and seven of them in different cities in the State of São Paulo. According to the QF residents, this community is descended from approximately 40 individuals from two families who remained in the region after the establishment of PESM. Although six of the 17 local experts were not born in the quilombola communities, they spent much of their lives living in these places and therefore suffered cultural interference from those. In this survey it was observed that the birth or upbringing of the local experts within the community can contribute to a closer relationship with their parents and relatives, and consequently, a greater learning about the knowledge of the local culture over the years.

So that local researchers could record the use of plants of their respective quilombos, they scheduled unstructured interviews (Bernard, 1988; Alexiades, 1996) each lasting about 60 min and repeated from 4 to 5 times with each interviewee for greater comprehensiveness and to check for additional knowledge to record. Interview forms were used both for socioeconomic data (name, sex, age, education and occupation), and for ethnobotanical data (such as the local name of the plant, part used, type of use and method of preparation). The university’s technical team (coauthors Sauini and Yazbek) accompanied them throughout these recording activities, supporting the work by taking notes in field diaries (Bernard, 1988) and observing participants, living in the communities and attending festivities and other day-to-day activities, such as caring for the fields and their businesses (restaurants), as well as making handicrafts. The plants indicated during the interviews were collected with monitoring by the interviewees of the drying method (Alexiades, 1996) and following a collection form used by the CEE (Center for Ethnobotanical and Ethnopharmacological Studies); later, they were pressed, dried and taken to the Municipal Herbarium (PMSP) for identification and deposition. For more details on the methodology, see previously published articles derived from this study (Rodrigues et al., 2020; Yazbek et al., 2019; Sauini et al., 2020). After being identified, plant species were searched in botanical databases to determine which were native and which were introduced to the Atlantic Forest.

It is worth mentioning that this participatory research had as advantages the fact that: (i) considering that the work was conducted with the active participation of the residents, its final result reflects the perceptions of each of the individuals involved, who were collectively responsible for its realization; (ii) local researchers have appropriated themselves of their traditional knowledge during the interviews and records conducted, increasing their role in decision-making on how to use available resources in that environment; (iii) the selection of plant experts to be interviewed has been made by local researchers, expanding the possibility of exhausting the register of knowledge of those communities; since no one better than the members of a particular community to know who the real local actors are; (iv) the participants have demonstrated several aspects about how they see and perform conservation of their environment; which they wanted to leave recorded through the audiovisuals; still (v) the young people of the community began to value the knowledge of their “fathers and mothers”; once they understand that this knowledge, collected by the residents themselves, favor the maintenance of their culture.

Among the disadvantages of this method, one can highlight: (i) the fact that few people were actually involved in the research, although the entire community has been consulted and invited to participate. One of the factors that may be associated with this situation was the delay in the beginning of the research that took about a year for all authorizations to be approved by the various Brazilian agencies. A woman from the Quilombo da Fazenda, for example, participated in the initial meetings for the development of the project and showed interest in being a local researcher, but when the fieldwork in fact began her son was about to be born. The same occurred with other people who received job offers and other activities that ended up making their participation unfeasible; still (ii) although it has not been observed in this work; it is important to reflect that in a participatory research there must be care to ensure that all local experts in the community are selected and/or provide their knowledges, even if there is any personal conflict between the local researcher and them, otherwise this may be an unfavorable factor to record as close as possible the knowledge of that community.

The participatory ethnobotany methods utilized in this study incorporated the needs of the communities. Thus, the communities defined the objectives of the study to register their knowledge, which are going through a process of transformation and loss of their culture, due to various factors such as: (i) construction of a highway close to the communities, which facilitated access to the areas; (ii) the departure of some young people from the areas to the city, with no interest in continuing local practices; finally (iii) the death of the elders, who were highly knowledgeable about the local culture, mainly the two oldest community leaders (Mr. Zé Pedro and Mr. Genésio) who died during this study. They actively participated in the process of building this survey, as well as immortalizing their knowledge about the uses of plants, animals and aspects of conservation, through booklet and audiovisual records. It was also observed that with the dissemination of these records, young people, children and even some adults made comments valuing the local experts, such as: “Wow, look at my grandpa, how important” (pointing to the booklet). And they even learned more about their culture, for example, during one of the workshops held, the most experienced “canoe builder” in the community taught our team and local children about the techniques of building small canoes, used as crafts (Thamara Sauini, 29 October 2017, personal observation).

### Diversity of local species: comparison between quilombos

To test whether there were differences between the quilombos in the proportions of plants that were native species or introduced, a 2 × 2 contingency table was created, and a chi-square test was applied.

To assess whether there were differences in the composition of known species between the quilombos, the data on the mentioned species were initially converted into a Jaccard dissimilarity matrix. Then, a permutational multivariate analysis of variance (PERMANOVA) was used to compare the dissimilarities in knowledge between quilombos; for this purpose, the adonis2 function of the vegan package was used (Oksanen et al., 2019), using 999 permutations. This procedure was performed for all categories of use studied, with the exception of the category of “combustion” because in one of the quilombos, only two individuals mentioned species for this category.

Subsequently, a multivariate dispersion homogeneity test (PERMDISP2) was used to assess whether the beta diversity (variation in the composition of species known among individuals) differed between quilombos. This analysis calculates the centroid of specific group (in our case, the people in each of the quilombos), and compares the average dissimilarity of the N individual observations of that group (the repertoire of species known to each of the local experts) using a measure of distance (in our case, Jaccard’s dissimilarity) (Anderson, 2006). Thus, if cultural and environmental differences determine the differences in species known between quilombos, low dissimilarity in local knowledge among individuals in each quilombo would be expected. It was performed this analysis using the betadisper function of the vegan package (Oksanen et al., 2019).

Finally, to visualize the patterns of dissimilarity in local knowledge, it was performed nonmetric multidimensional scaling (nMDS). In all cases, the stress of the nMDS graph was ≤0.1, indicating that the two-dimensional graph was sufficient to represent clearly the differences between the sampling units. It was designed all the charts using the ggplot2 (Wickham, 2016) and ggpubr (Kassambara, 2020) packages. Also it was performed all analyses in the R environment (R Core Team, 2020). The three categories: “handicrafts”, “shipbuilding” and “technology” were merged into the “fibers in technology” category, since individually they had low number of ethnobotanical indications, making impossible the statistical analyzes necessary for this study.

## Results and discussion

### Analysis of the diversity of species known to local experts: comparison between quilombos

**Figure 2:**
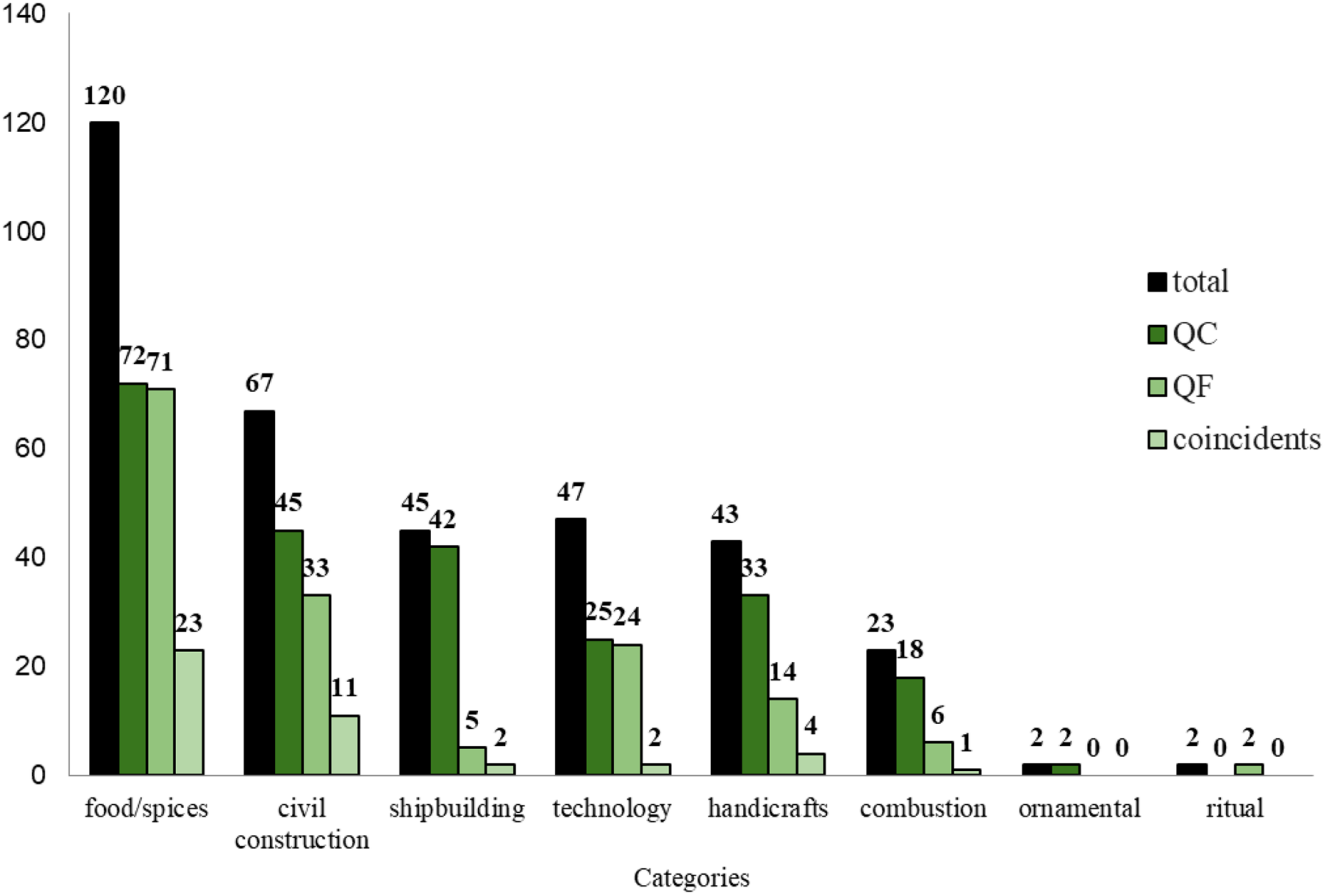
Number of species indicated by the 17 local experts from Quilombo da Fazenda (QF) and Quilombo do Cambury (QC), for the eight categories of uses. Total data of both quilombos; per quilombo; and coincident data between quilombos.

A total of 214 plant species were indicated by the 17 local experts in both quilombos; and they were grouped into eight categories of use, according to the classification used by Galleano (Galleano, 2016): “ornamental” (two species), “food/spices” (120), “technology” (47), “combustion” (23), “ritual” (2), “civil construction” (67), “shipbuilding” (45) and “handicrafts” (43), see Table 1 and Fig. 2. The same species can be present in more than one category simultaneously. The “medicinal” category, although documented in the present study, will be the subject of another publication due to its abundance of data, deserving deep and robust analyses. Table 1 presents the 214 plant species according to their families, voucher numbers, scientific and popular names, uses and parts used in both quilombos.

**Table 1:** A total of 214 plant species belonging to the eight categories of use, indicated by the 17 local experts living in Quilombos do Cambury (QC) and Fazenda (QF); their families, voucher numbers, scientific and popular names, uses and parts used.

**Family**	**Scientific name (Voucher)**	**Popular name**	**Uses (emic terms)**	**Used part**	**Quilombo**
**Category “Food/Spices”**					
Amaranthaceae	*Amaranthus blitum* L.—PBY86	cururu or caruru	refogar e comer (saute and eat)	fo	QF
Amaryllidaceae	*Allium chinense* G. Don—CC021	alho	tempero (spice)	fo	QC
	*Allium fistulosum* L.—CC 012	cebolinha	tempero (spice)	fo	QC
	*Allium tuberosum* Rottler ex Spreng.—CC013	alho-de-folha	tempero (spice)	fo	QC
Anacardiaceae	*Anacardium occidentale* L.—GDS14*	caju	comer o fruto (eat the fruit)	fr	QF
	*Schinus terebinthifolia* Raddi—SB17*	aroeira	tempero (spice)	fr	QF
Annonaceae	*Annona dolabripetala* Raddi—SB52*	roque or São-Roque	comer o fruto (eat the fruit)	fr	QF
	*Annona montana* Macf.—THS 149*	graviola	alimento (food)	fr	QC
	*Annona mucosa* Jacq. GDS21*	condessa	comer o fruto, fazer suco (eat the fruit, make juice)	fr	QF
	*Annona muricata* L.—GDS52	graviola	comer o fruto (eat the fruit)	fr	QF
Apiaceae	*Coriandrum sativum* L.—SB24	coentro-miúdo	tempero (spice)	fo	QF
	*Eryngium* cf. *coronatum* Hook. & Arn.—CC022	coentro-de-folha-comprida	tempero (spice)	fo	QC
		coentro	tempero (spice)	fo	QF
	*Eryngium foetidum* L.—CC 009*	coentro	tempero (spice)	fo	QC
		coentro-natural, gigante or caiçara	tempero (spice)	fo	QC
	*Foeniculum vulgare* Mill.—CC25	erva-doce	fazer chá (make tea)	pt	QF
Aracaceae	*Bactris gasipaes Kunth—*SB58	pupunha	comer o fruto e o palmito (eat the fruit and the palm heart)	fr; map	QF
	*Euterpe oleracea* Mart.—PBY54	açaí	comer o fruto e o palmito (eat the fruit and the palm heart)	fr; map	QF
	*Syagrus pseudococos* (Raddi) Glassman—PBY70*	patieiro	comer o palmito da palmeira nova; comer o palmito da palmeira ainda brotando; comer palmito (eat the palm heart of the new palm tree; eat the palm heart of the palm still sprouting; eat palm heart)	map	QF
Araceae	*Colocasia esculenta* (L.) Schott—SB11	inhame	cozinhar o tubérculo e comer; cozinhar a raiz e comer (to cook the tuber and eat; cook the root and eat)	tu	QF
	*Xanthosoma taioba E.G*.Gonç.—GDS20*	taioba	refogar ou cozinhar as folhas e comer; salada com folhas e comer raiz (sauté or cook the leaves and eat; salad with leaves and root)	fo	QF
Arecaceae	*Astrocaryum aculeatissimum* (Schott) Burret—MAS 049*	coco-preto or coco-bejaúva	alimento (food)	fr	QC
	*Bactris* sp.—MAS 046	coco-mirim	alimento (food)	fr	QC
	*Euterpe edulis* Mart.—GDS 27*	palmito-jussara	alimento (food)	fr	QC
		juçara	comer o fruto e o palmito (eat the fruit and the palm heart)	fr; map	QF
Asteraceae	*Achillea millefolium* L.—MAS 001	novalgina	tempero (spice)	fo	QC
	*Baccharis* sp. (Sect. Caulopterae DC.)—PBY74	carqueja	colocar na pinga (put in the drip)	fo	QF
	*Elephantopus mollis* Kunth—MA077*	erva-grossa	tempero de feijão (bean seasoning)	fo	QC
	*Emilia fosbergii* Nicolson—PBY45*	serralha-não-legítima	fazer salada (make salad)	fo	QF
	*Emilia sonchifolia* (L.) DC.—PBY105	serralha; serralha-branca	salada, cozinhar ou refogar e comer (salad, cooking or sautéing and eating)	fo	QF
	*Erechtites hieracifolius* (L.) Raf. ex DC.—PBY84*	serralha-roxa	comer como salada (eat like salad)	fo	QF
	*Erechtites valerianifolius* (Wolf) DC.—GDS08	gondó or caporoçova, serralha-roxa, serralha-branca	salada, refogar e comer (salad, sauté and eat)	fo; ca	QF
		capirosoba	salada (salad)	fo	QC
	*Hypochaeris chillensis* (Kunth) Britton—SB40*	almeirão-amargo or do mato	salada (salad)	fo	QF
	*Cichorium* cf. *intybus* L.—PBY41	almeirão-amargo or do mato	salada (salad)	fo	QF
	*Lactuca indica* L.—GDS39	almeirão-japonês or roxo	salada (salad)	fo	QF
Bignoniaceae	*Jacaranda puberula* Cham.—MA056*	caroba-branca	alimento (food)	fr	QC
Bixaceae	*Bixa orellana* L.—TH071*	urucum	colorau (paprika)	fr	QC
	*Brassica oleracea* L. (Gr. Acephala)—SB39	couve-dura, couve-mole or manteiga	salada, refogado (salad, stir-fry)	fo	QF
Caricaceae	*Jacaratia spinosa* (Aubl.) A.DC.—MA112*	mamão-do-mato	alimento (food)	fr	QC
Chrysobalanaceae	*Licania* sp.2—MA051	milho-torrado	alimento (food)	fr	QC
Clusiaceae	*Garcinia gardneriana* (Planch. & Triana) Zappi—TH153*	bacubari	alimento (food)	fr	QC
Convolvulaceae	*Ipomoea batatas* (L.) Lam.—MA088	batata-doce	alimento (food)	tu	QC
Cucurbitaceae	*Cucurbita* cf. *maxima* Duchesne—SB41	abóbora	cozinhar ou refogar e comer (salad, re-cook or sauté and eat steamed)	fr	QF
Dioscoreaceae	*Dioscorea bulbifera* L.—GDS45	cará-branca or caramoela	cozinhar o tubérculo e comer (cook the tuber and eat)	tu	QF
	*Dioscorea* sp.—MA068	cara-espinho	alimento (food)	tu	QC
Elaeocarpaceae	*Elaeocarpus* cf. *serratus* L.—MA032	azeitona-de-zeilão	fazer doces e conservas (make sweets and preserves)	fr	QC
Euphorbiaceae	*Aleurites moluccanus* (L.) Willd.—MA137	angora	cozinhar o fruto para tirar o óleo e usar para preparo de alimentos (cooking the fruit to remove the oil and use it for food preparation)	fr	QC
	*Mabea piriri* Aubl.—MA047*	cano-de-pito	alimento (food)	ca	QC
	*Manihot esculenta* Crantz—GDS46*	mandioca-doce, mandioca-brava	comer a raíz, fazer farinha (eat the root, make flour)	ra	QF
		mandioca-vermelhinha	alimento (food)	tu	QC
	*Tetrorchidium* sp.—MAS020	bapeva	alimento (food)	fr	QC
Fabaceae	*Hymenaea altissima* Ducke—SB49*	jatobá or jataí	tomar o vinho (drink the wine)	ex	QF
	*Inga edulis* Mart.—GDS72*	ingá-de-metro	comer o fruto (eat the fruit)	fr	QF
	*Inga marginata* Willd.—GDS44*	ingá-feijão	comer o fruto (eat the fruit)	fr	QF
	*Cajanus cajan* (L.) Huth—THS072	feijão-guandú	alimento (food)	fr	QC
	cf. *Swartzia oblata* R.S.Cowan—MA098*	jatobá	alimento (food)	fr	QC
	*Hymenaea* cf. *altissima* Ducke—THS132*	jatobá	alimento e bebida com o vinho que sai de seu tronco (food and drink with the wine that comes out of its trunk)	fr	QC
	*Inga* cf. *lenticellata* Benth.—MA027*	ingá-ferro	fazer doces (make candy)	fr	QC
	*Inga marginata* Willd.—MA070*	ingá-feijão	alimento (food)	fr	QC
	*Phaseolus vulgaris* L.—MA026	feijão	alimento (food)	fr	QC
Lamiaceae	*Mentha* sp.—SB61	hortelã-de-bicha	tempero (spice)	fo	QF
	*Ocimum americanum* L.—CC011	manjericão	alimento (food)	fo	QC
		manjericão	tempero (spice)	fo	QF
	*Ocimum campechianum* Mill.—CC010	favaca, alfavaca	tempero (spice)	fo	QC
		alfavaca	tempero (spice)	fo	QF
	*Ocimum gratissimum* L.—CC014	favacão	tempero (spice)	fo	QC
		favacão	tempero (spice)	fo	QF
	*Plectranthus amboinicus* (Lour.) Spreng.—SB32	hortelã-castelo or hortelã-de-carne	tempero (spice)	fo	QF
Lauraceae	*Cryptocarya mandioccana* Meisn.—PBY20	noz-moscada	alimento (food)	fr	QC
		noz-moscada	colocar na pinga, usar para temperar e fazer bolo (put in the pinga, use to season and make cake)	se	QF
	*Cryptocarya saligna* Mez—PBY24*	canela-sassafraize	colocar na pinga (put in the drip)	cs	QF
	*Nectandra oppositifolia* Nees—MA103*	caneleira	alimento (food)	fr	QC
	*Persea americana* Mill.—GDS004	abacate	alimento (food)	fr	QC
Malpighiaceae	*Bunchosia glandulifera* (Jacq.) Kunth—GDS56	guaraná or cerejinha	comida de passarinho, comer o fruto (bird food, eat the fruit)	fr	QF
	*Malpighia glabra* L.—GDS67	acerola	comer o fruto, fazer a polpa (eat the fruit, make the pulp)	fr	QF
Malvaceae	*Abelmoschus esculentus* (L.) Moench—PBY94	quiabo	comer o fruto (eat the fruit)	fr	QF
	*Pachira glabra* Pasq.—PBY02	castanha	comer o fruto (eat the fruit)	fr	QF
	*Theobroma cacao* L.—SB45	cacao	Comer o fruto ou secar as sementes no sol, torrar e moer para fazer achocolatado (eat the fruit or dry the seeds in the sun, roast and grind to make chocolate milk)	fr	QF
	*Theobroma cacao* L.—THS017	cacao	alimento (food)	fr	QC
Marantaceae	*Marantaceae* sp.2—THS164	araruta	fazer farinha (make flour)	pt	QC
Melastomataceae	*Clidemia hirta* (L.) D.Don—MA073*	pixirica	alimento (food)	fr	QC
		pixirica, guanum or maria pretinha	comer o fruto (eat the fruit)	fr	QF
	*Miconia dodecandra* Cogn.—MA138*	pixirica	alimento (food)	fr	QC
	*Miconia prasina* (Sw.) DC.—THS067*	pixirica	alimento (food)	fr	QC
Meliaceae	*Trichilia silvatica* C.DC.—MAS058*	pixiricão	alimento (food)	fr	QC
Moraceae	*Artocarpus altilis* (Parkinson) Fosberg—SB74	fruta-pão	cozinhar o fruto e comer com manteiga (cook the fruit and eat it with butter)	fr	QF
	*Artocarpus heterophyllus* Lam.—MAS084	jaqueira	alimento (food)	fr	QC
	*Morus nigra* L.—GDS068	amora	alimento (food)	fr	QC
		amora, amora-branca	comer o fruto, fazer polpa (eat the fruit, make pulp)	fr	QF
Musaceae	*Musa* sp.—PBY069	bananeira	alimento (food)	fr	QC
Myrtaceae	*Campomanesia phaea* (O.Berg) Landrum—GDS33*	cambuci	comer o fruto, fazer a polpa e fazer doces como geléia (eat the fruit, make the pulp and make sweets like jelly)	fr	QF
		cambuci	alimento (food)	fr	QC
	*Eugenia brasiliensis* Lam.—SB55*	grumixama	comer o fruto (eat the fruit)	fr	QF
	Eugenia cf. multicostata D. Legrand—THS032*	carambola-do-mato; pau-Brasil	alimento (food)	fr	QC
	*Eugenia* cf. *stipitata* McVaugh—GDS73	araçá-do-norte, de morcego or cerrado	comer o fruto (eat the fruit)	fr	QF
	*Eugenia* sp.—THS131	goiabinha	alimento (food)	fr	QC
	*Eugenia sulcata* Spring ex Mart.—MA069*	pitanga-do-mato	alimento (food)	fr	QC
	*Eugenia uniflora* L.—CC007*	pitanga	alimento (food)	fr	QC
		pitanga	comer o fruto (eat the fruit)	fo	QF
	*Myrcia neoriedeliana* E.Lucas & C.E.Wilson—PBY26*	cambucá-do-mato	comer o fruto (eat the fruit)	fr	QF
	*Myrcia spectabilis* DC.—THS060*	arueira	alimento (food)	fr	QC
	*Myrciaria glazioviana* (Kiaersk.) G.M.Barroso ex Sobral—GDS15*	cabeludinha	comer o fruto (eat the fruit)	fr	QF
		cabeludinha	alimento (food)	fr	QC
	*Plinia edulis* (Vell.) Sobral—MA100*	cambucá	alimento (food)	fr	QC
		cambucá	comer o fruto (eat the fruit)	fr	QF
	*Plinia* sp.—MA101	jaboticaba	alimento (food)	fr	QC
	*Psidium cattleyanum* Sabine—MA107*	aracá	alimento (food)	fr	QC
		araçá-de-beira-de-praia	comer o fruto (eat the fruit)	fr	QF
	*Psidium guajava* L.—GDS34	goiaba	comer o fruto, fazer suco (eat the fruit, make juice)	fr	QF
		goiaba	alimento (food)	fr	QC
	*Syzygium jambos* (L.) Alston—GDS01	jambo-amarelo	comer o fruto (eat the fruit)	fr	QF
	*Syzygium malaccense* (L.) Merr. & L.M.Perry—GDS01	jambo, jambo-roxo or jambolão	comer o fruto (eat the fruit)	fr	QF
		jambo	alimento (food)	fr	QC
Passifloraceae	*Passiflora miersii* Mast.—THS108*	maracujá-do-mato	alimento (food)	fr	QC
Plantaginaceae	*Plantago australis* Lam.—MA044*	tansagem	salada (salad)	fo	QC
		trançagem ou tanchagem	salada (salad)	fo	QF
Poaceae	*Cymbopogon citratus* (DC.) Stapf—SB69	capim-limão ou capim-cheiroso	fazer o chá (suco) (make juice)	fo	QF
Rosaceae	*Eriobotrya japonica* (Thunb.) Lindl.—PBY52	ameixa; ameixa-amarela	fazer o chá (suco) (make juice)	fr	QF
	*Rubus rosifolius* Sm.—SB62	amora	comer o fruto (eat the fruit)	fr	QF
		moranguinho-do-mato/amora	alimento (food)	fr	QC
	*Rubus urticifolius* Poir.—THS013*	amora-de-cacho	alimento (food)	fr	QC
Rubiaceae	*Coffea arabica* L.—SB14	café	torrar o fruto, para fazer café (roast the fruit, to make coffee)	fr	QC
		café, café-gigante	pó de café (coffee powder)	fr	QF
Rutaceae	*Citrus reticulata* Blanco—GDS69	laranja, mixirica	comer o fruto, fazer a polpa (eat the fruit, make the pulp)	fr	QF
	*Citrus sinensis* (L.) Osbeck—GDS03	laranjeira	alimento (food)	fr	QC
	*Citrus x limon* (L.) Osbeck—GDS40	limão	temperar, fazer a polpa (spice)	fr	QF
		limão	alimento (food)	fr	QC
Sapotaceae	*Capsicum* sp.—PBY104	pimento-chifre-de-veado	comer o fruto (eat the fruit)	fr	QF
	C*apsicum* sp.—SB33	pimento-doce	temperar a comida (season the food)	fr	QF
	*Ecclinusa ramiflora* Mart.—THS154*	guacuá	alimento (food)	fr	QC
	*Micropholis crassipedicellata* (Mart. & Eichler) Pierre—MAS053*	bacubixaba	alimento (food)	fr	QC
	*Mimusops coriacea* (A.DC.) Miq.—THS052	abricó	fazer doce com o fruto (make candy with the fruit)	fr	QC
	*Pouteria caimito* (Ruiz & Pav.) Radlk.—SB67*	abiu	comer o fruto (eat the fruit)	fr	QF
	*Solanum americanum* Mill.—SB23*	erva-moura	Salada; Comer refogado para não “picar”, pegar na garganta (salad and it is recommended to eat sautéed so as not to hurt the throat)	fo	QF
Solanaceae	*Solanum pseudoquina* A.St.-Hil.—MA134*	pilotera	alimento (food)	fr	QC
	*Solanum scuticum* M.Nee—MAS030*	jurubeba	fazer conservas (preserve)	fr	QC
Urticaceae	*Cecropia glaziovii* Snethl.—PBY68*	embaúba	cozinhar ou refogar e comer (cook or sauté and eat)	fr	QF
	*Cecropia pachystachya* Trécul—PBY 22*	embaúba	cozinhar ou refogar e comer (cook or sauté and eat)	fr	QF
	*Urera nitida* (Vell.) P.Brack—MA095*	urtiga	salada (salad)	fo	QC
	*Urera baccifera* (L.) Gaudich. ex Wedd.—GDS07*	urtiga-roxa	salada (salad)	fo	QF
Verbenaceae	*Citharexylum myrianthum* Cham.—THS010*	tarumã	alimento (food)	fr	QC
Zingiberaceae	*Curcuma longa* L.—PBY67	açafrão	temperar comida (season the food)	ra	QF
**Category “Civil Construction”**					
Annonaceae	*Xylopia brasiliensis* Spreng.—MAS031*	canafista	construção de casas (build houses)	ca	QC
Araceae	*Philodendron eximium* Schott—GDS29*	imbé	construir casas (build houses)	li	QF
Araliaceae	*Schefflera* cf. *angustissima* (Marchal) Frodin—MA116*	imbirotó	construção de casas (house building)	ca	QC
Arecaceae	*Euterpe edulis* Mart.—GDS27*	juçara	construir casas (ripas) (build houses laths)	ca	QF
	*Geonoma elegans* Mart.—SB51	guaricanga	esteira para telhado (roof mat)	fo	QF
	*Geonoma* sp.—MA122	urecanga	construção de telhados (roof construction)	fo	QC
	*Syagrus pseudococos* (Raddi) Glassman—PBY70*	patieiro e patiuava	esteio, casas e capelo da casa de sapê (mainstay, houses and capel of the thatched house)	ca; fo; ca	QF
Bignoniaceae	*Aniba* sp.—THS09	loro	táboas (boards)	ca	QF
	*Handroanthus albus* (Cham.) Mattos—PBY090*	ipê-amarelo	construção de casas (house building)	ca	QC
		ipê amarelo	construir casas: esteio, caibo e ripa (build houses: stanchion, rafter and batten)	ca	QF
	*Jacaranda puberula* Cham.—MA056*	carobinha	construção de casas (house building)	ca	QC
Boraginaceae	*Cordia* sp.2—THS080	louro-pardo	construção de casas (house building)	ca	QC
Chloranthaceae	*Hedyosmum brasiliense* Miq.—THS119*	congonha	construção de casas (house building)	ca	QC
Chrysobalanaceae	*Licania* sp.2—MAS051	milho-torrado	construção de casas (house building)	ca	QC
Clusiaceae	*Garcinia gardneriana* (Planch. & Triana) Zappi—PBY21*	bacupari	construir casas, travessa (build houses)	ca	QF
Cyclanthaceae	cf. *Thoracocarpus bissectus* (Vell.) Harling—SB18*	timupeva	construção de casas (house building)	li	QF
Erythroxylaceae	*Erythroxylum pulchrum* A.St.-Hil.—PBY04*	guará-cipó	construir casas (build houses)	ca	QF
Euphorbiaceae	*Actinostemon verticillatus* (Klotzsch) Baill.—MAS043*	sucanga	construção de casas (house building)	ca	QC
	*Mabea piriri* Aubl.—MA047*	cano-de-pito, canudo-de-pito	construção de casas (house building)	ca	QC
		canudo-de-pito	construir casas (build houses)	ca	QF
	*Tetrorchidium* sp.—THS062	bapeva	construção de casas (house building)	ca	QC
Fabaceae	*Andira fraxinifolia* Benth.—MA102*	sucupira	construção de casas (house building)	ca	QC
	cf. *Hymenolobium janeirense* Kuhlm.—MA060*	guacuí	construção de casas (house building)	ca	QC
	cf. *Pterocarpus rohrii Vahl—*PBY07*	guaricica or guaricica-amarela	construir casas: travessa e caibro (build houses: transom and rafter)	ca	QF
	*Inga* cf. *lenticellata* Benth.—MAS027*	ingá-ferro	construção de casas (house building)	ca	QC
	*Inga* sp.—MAS048	ingá-macaco	construção de casas (house building)	ca	QC
	*Myrocarpus frondosus* Allemão—GDS53*	cabreúva	construir casas (esteios) (build houses)	ca	QF
	*Piptadenia gonoacantha* (Mart.) J.F.Macbr.—GDS09*	caniveteiro	construir casas: esteio e tábuas (build houses: mainstay and boards)	ca	QF
	*Swartzia simplex* var.grandiflora (Raddi) R.S.Cowan—MAS 047*	laranjeira-do-mato	construção de casas (house building)	ca	QC
		canela-prego	construir casas: esteio, viga e caibo, e móveis (build houses: mainstay, beam and rafter, and furniture)	ca	QF
	*Tachigali paratyensis* (Vell.) H.C.Lima—MAS129*	ingá-flecha	construção de casas (house building)	ca	QC
	*Tachigali* sp.1—MA055	ingá-amarelo	construção de casas (house building)	ca	QC
	*Tachigali* sp.2—MAS021	ingá-flecha	construção de casas (house building)	ca	QC
	cf. *Hymenolobium janeirense* Kuhlm.—MA060*	guiti	construção de casas (house building)	ca	QC
Lacistemataceae	*Lacistema lucidum* Schnizl—MAS040*	tatuzinho	construção de casas (house building)	ca	QC
		burrachudo	construir casas: travessa e caibo (build houses: lane and rafter)	ca	QF
Lamiaceae	*Vitex polygama* Cham.—THS010*	tarumã	construção de casas (house building)	ca	QC
Lauraceae	*Aniba* sp.—THS103	canela-parda or amarela	construir casas, esteios (build houses, mainstays)	ca	QF
		canela-do-mato	construção de casas (house building)	ca	QC
	*Cryptocarya saligna* Mez—PBY24*	canela-sassafraize	construir casas: esteio, caibro e travessa, e móveis (build houses: mainstay, rafter and transom, and furniture)	ca	QF
	*Nectandra oppositifolia* Nees—MA103*	caneleira (canela-do-mato)	construção de casas (house building)	ca	QC
Lecythidaceae	*Cariniana estrellensis* (Raddi) Kuntze—MAS038*	jequitibá	construção de casas (house building)	ca	QC
Malvaceae	*Eriotheca pentaphylla* (Vell. & K.Schum.) A.Robyns—MAS044*	imbiruçú	construção de casas (house building)	ca	QC
Melastomataceae	*Miconia cinnamomifolia* (DC.) Naudin—GDS50*	jacatirão	construir casas: travessas e caibros (build houses: sleepers and rafters)	ca	QF
		jacatirão	construção de casas (house building)	ca	QC
	*Tibouchina pulchra* Cogn.—MA110*	quaresmeira	construção de casas (house building)	ca	QC
Meliaceae	*Cabralea canjerana* (Vell.) Mart.—MAS052*	ingá-cajarana	construção de casas (house building)	ca	QC
	*Cedrela fissilis* Vell.—SB34*	cedro-rosa	construir casas (build houses)	ca	QF
	*Trichilia silvatica* C.DC.—MAS054*	pixiricão	construção de casas (house building)	ca	QC
Moraceae	*Brosimum guianense* (Aubl.) Huber—THS043*	guaricica-da-vermelha	construção de casas (house building)	ca	QC
	*Sorocea* cf. *guilleminiana* Gaudich.—PBY09*	garapinha	esteio (mainstay)	ca	QF
		espinheira-Santa	construção de casas (house building)	ca	QC
Myristicaceae	*Virola bicuhyba* (Schott ex Spreng.) Warb.—MA113*	bicuíba	construção de casas (house building)	ca	QC
Myrtaceae	*Campomanesia phaea* (O.Berg.) Landrum—THS161*	cambuci	construção de casas (house building)	ca	QC
	*Eugenia* cf. *multicostata* D.Legrand—THS032*	carambola-do-mato	construção de casas (house building)	ca	QC
	*Eugenia* cf. *stipitata* McVaugh—GDS73	araça-do-norte or de morcego	construir casas: esteio, travessa e caibo (build houses: mainstay, crossbeam and rafter)	ca	QF
	*Myrcia spectabilis* DC.—PBY05*	arco-de-peneira	construir casas: travessa (build houses: alley)	ca	QF
	sp. 2—PBY12	muta	construir casas: caibro (build houses: rafter)	ca	QF
Peraceae	*Pera glabrata* (Schott) Poepp. ex Baill.—GDS47*	chile	construir casas: travessa e caibro (build houses: transom and rafter)	ca	QF
Phyllanthaceae	*Hyeronima alchorneoides* Allemão—MAS035	aricurana	construção de casas (house building)	ca	QC
		aricurana	construir casas: esteio, batente de porta, caibo e viga e tábua (build houses: mainstay, door frame, rafter and beam and board)	ca	QF
Poaceae	*Bambusa* cf. *vulgaris* Schrad. ex J.C.Wendl.—GDS26	bambu-gigante	construir casas: varas (build houses: sticks)	ca	QF
	*Bambusa* sp.—SB027	bambú	construção de casas (house building)	ca	QC
	*Bambusa* sp.—SB27	bambu	construir casas: varas e para amarrar os pilares maiores (build houses: sticks and to tie the larger pillars)	ca	QF
	*Imperata* sp.—GDS71	sapê	esteira para telhado (roof mat)	fo	QF
Primulaceae	*Myrsine coriacea* (Sw.) R.Br. ex Roem. & Schult.—MA139*	capuroroca	construção de casas (house building)	ca	QC
		capororoca	construir casas: travessa (build houses: alley)	ca	QF
	*Stylogyne lhotzkyana* (A.DC.) Mez—THS122*	sapopema	construção de casas (house building)	ca	QC
Rubiaceae	*Bathysa mendoncaei* K.Schum.—BY15*	sapopema	construir casas: caibro e travessa (build houses: rafter and crossbeam)	ca	QF
	cf. *Bathysa—*PBY14	aribarrosa	construir casas: caibro e travessa (build houses: rafter and crossbeam)	ca	QF
	*Rustia formosa* (Cham. & Schltdl.) Klotzsch—THS175*	manduberana	construção de casas (house building)	ca	QC
Sapindaceae	*Cupania oblongifolia* Mart.—THS148*	cubatam	construção de casas, telhado (house building roof)	ca	QC
		cubatã	construir casas: travessa (build houses: alley)	ca	QF
Sapotaceae	*Micropholis crassipedicellata* (Mart. & Eichler) Pierre—MAS053*	bacubixaba	viga, caibro, ripa (beam, rafter, batten)	ca	QC
	*Pouteria caimito* (Ruiz & Pav.) Radlk.—THS07*	guapeva	construção de casas: caibros e travessas (construction of houses: rafters and sleepers)	ca	QF
	*Pouteria* sp.2—THS063	guacuáuçu	construção de casas (house building)	ca	QC
Urticaceae	*Cecropia glaziovii* Snethl.—THS178*	embaúba	construção de casas (house building)	ca	QC
Verbenaceae	*Citharexylum myrianthum* Cham.—THS010*	tarumã	construção de casas (house building)	ca	QC
**Category “Combustion”**					
Cannabaceae	*Trema micranta* (L.) Blume—THS157*	gandiúba; candiúba	Madeira, pólvora, combustão (Wood, gunpowder, combustion)	ca	QC
Euphorbiaceae	*Actinostemon verticillatus* (Klotzsch) Baill.—MAS043*	sucanga	Lenha, madeira (firewood, wood)	ca	QC
	*Aleurites moluccanus* (L.) Willd.—MA137	angora	cozinhar o fruto para tirar o óleo (cook the fruit to get the oil out)	fr	QC
	*Mabea piriri* Aubl.—MA047*	cano-de-pito	lenha (firewood)	ca	QC
	*Ricinus communis* L.—GDS51	mamona	óleo retirado do fruto era usados para pôr no candeeiro (oil taken from the fruit was used to put on the lamp, a type of lampshade)	fr	QF
Fabaceae	*Swartzia simplex* var. grandiflora (Raddi) R.S.Cowan—PBY08*	laranjeira-do-mato	lenha (firewood)	ca	QF
	*Dalbergia frutescens* (Vell.) Britton—MA054*	braço-forte	lenha (firewood)	ca	QC
	*Hymenaea* cf. *altissima* Ducke—MAS009*	jatobá	óleo (oil)	fr	QC
	*Swartzia simplex* var.grandiflora (Raddi) R.S.Cowan—MAS047*	laranjeira-do-mato	lenha (firewood)	ca	QC
	*Tachigali paratyensis* (Vell.) H.C.Lima—MA0129*	ingá-flecha	lenha (firewood)	ca	QC
	*Tachigali* sp.2—MA055	ingá-flecha	fazer carvão (make charcoal)	ca	QC
Lacistemataceae	*Lacistema lucidum* Schnizl—MAS040*	tatuzinho	lenha (firewood)	ca	QC
Lamiaceae	*Aegiphila integrifolia* (Jacq.) Moldenke—THS11*	cajuja	óleo (oil)	ca	QF
Melastomataceae	*Tibouchina pulchra* Cogn.—MA110*	manacá-da-serra/quaresmeira	lenha (firewood)	ca	QC
Myristicaceae	*Virola bicuhyba* (Schott ex Spreng.) Warb.—MA113*	bicuíba	óleo, lenha (oil, firewood)	se	QC
Myrtaceae	*Eugenia astringens* Cambess.—PBY30*	araçarana	lenha (firewood)	ca	QF
	*Plinia edulis* (Vell.) Sobral—MA100*	cambucá	lenha (firewood)	ca	QC
Peraceae	*Pera glabrata* (Schott) Poepp. ex Baill.—GDS47*	chile	lenha (firewood)	ca	QF
		casca-preta, Chile	lenha (firewood)	ca	QC
Primulaceae	*Stylogyne lhotzkyana* (A.DC.) Mez—THS122*	sapopema	lenha (firewood)	ca	QC
Sapindaceae	*Cupania oblongifolia* Mart.—MA137*	angora	óleo do fruto (fruit oil)	fr	QC
Sapotaceae	*Ecclinusa ramiflora* Mart.—THS154*	guacá	lenha (firewood)	ca	QC
	*Pouteria caimito* (Ruiz & Pav.) Radlk.—THS07*	guapeva	lenha (firewood)	ca	QF
Solanaceae	*Solanum pseudoquina* A.St.-Hil.—MA134*	pilotera	lenha (firewood)	ca	QC
**Category “Handicrafts”**					
Annonaceae	*Annona dolabripetala* Raddi—MAS012*	araticum	artesanato, como tartarugas, remo, barquinhos (handicrafts such as turtles, rowing, boats)	ca	QC
Apocynaceae	*Malouetia cestroides* (Nees ex Mart.) Müll. Arg.–MAS034*	guairana	artesanato (handicrafts)	ca	QC
	*Tabernaemontana laeta* Mart.—SB54*	guarana	balaios e outros artesanatos (baskets and other handicrafts)	ca	QF
Araceae	*Philodendron eximium* Schott—GDS29*	imbé	balaios, cestas e outros artesanatos (baskets, baskets and other handicrafts)	li	QF
Araliaceae	*Schefflera* cf. *angustissima* (Marchal) Frodin—MA116*	imbirotó	artesanatos como tartarugas e canoinha (handcrafts like turtles and canoe)	ca	QC
Arecaceae	*Astrocaryum aculeatissimum* (Schott) Burret—GDS74*	brejaúba	balarios, cestas (baskets)	ca	QF
		coco-preto/bejaúva	anel de coco (coconut ring)	fr	QC
	*Bactris* sp. —MAS046	coco-mirim	anel de coco (coconut ring)	fr	QC
	*Euterpe edulis* Mart.—GDS27*	juçara	balarios, cestas (baskets)	fl; ca	QF
	*Geonoma* sp.—MA122	urecanga	artesanato (handicrafts)	ca	QC
	*Syagrus pseudococos* (Raddi) Glassman—PBY70*	patieiro	fruteiras, luminárias, balaios, cestas (fruit bowls, lamps and baskets)	bra	QF
Bignoniaceae	*Tabebuia cassinoides* (Lam.) DC.—GDS41*	caxeta	canoas, colher de pau, engenho pequeno (canoes, wooden spoon, small handicrafts)	ca	QF
Celastraceae	*Maytenus ardisiaefolia* Reissek—MAS022*	guaracipó	artesanato (handicrafts)	ca	QC
Clusiaceae	*Clusia criuva* subsp. parviflora Vesque—MA105*	mangue	artesanato (handicrafts)	ca	QC
Cucurbitaceae	*Lagenaria siceraria* (Molina) Standl.—PBY37	cabaça	instrumentos musicais (musical instruments)	fr	QF
Cyclanthaceae	cf. *Thoracocarpus bissectus* (Vell.) Harling—SB18*	timupeva	balaios e outros artesanatos (baskets and other handicrafts)	li	QF
Dilleniaceae	*Davilla rugosa* Poir.—MA035*	cipó-cabloco	mãos e pernas da lagostinha, suporte para apoiar a arara (handicrafts: crayfish hands and legs, support to support the macaw)	ca	QC
Euphorbiaceae	*Actinostemon verticillatus* (Klotzsch) Baill.—MAS043*	sucanga	artesanato (handicrafts)	ca	QC
	*Mabea piriri* Aubl.—MA047*	cano-de-pito	artesanato (handicrafts)	ca	QC
Fabaceae	*Inga edulis* Mart.—GDS72*	ingá-de-metro	balarios, cestas (baskets)	ca	QF
	cf. *Hymenolobium janeirense* Kuhlm.—MA060*	guacuí	artesanato (handicrafts)	ca	QC
Lamiaceae	*Aegiphila integrifolia* (Jacq.) Moldenke—THS11*	cajuja	balarios, cestas (baskets)	ca	QF
Lauraceae	*Nectandra oppositifolia* Nees—MA103*	canela-do-mato	tábua, barco, esteio (board, boat, mainstay)	ca	QC
Malvaceae	*Eriotheca pentaphylla* (Vell. & K.Schum.) A.Robyns—MAS044*	imbiruçú	artesanato (handicrafts)	ca	QC
	*Sida planicaulis* Cav.—THS086*	vassoura-chata	vassoura (broom)	fo	QC
	*Sida rhombifolia* L.—GDS19*	vassoura	fazer vassouras de enfeite (make decorative brooms)	pt	QF
		vassoura (qualidade 1)	vassoura (broom)	fo	QC
Meliaceae	*Guarea macrophylla* Vahl—SB53*	café-do-mato	arco, flecha artesanato e berimbau (bow, arrow craft and berimbau, a string and wooden instrument)	ca	QF
Moraceae	*Ficus adhatodifolia* Schott—MA121*	figueira-branca	gamela (trough, a wooden bowl)	ca	QC
	*Sorocea guilleminiana* Gaudich.—THS171*	guaricica-da-marrom	artesanato (handicrafts)	ca	QC
Myristicaceae	*Virola bicuhyba* (Schott ex Spreng.) Warb.—MA113*	bicuíba	gamela, banco (trough, a wooden bowl, and boat)	ca	QC
Myrtaceae	*Myrcia* sp.2—MA050	arco-de-peneira	arco da peneira, arco, bodoque, berimbau (sieve bow, bow, bodoque - like a slingshot, and berimbau, a string and wooden instrument)	ca	QC
	*Myrcia* sp.3—THS045	arco-de-peneira	arco da peneira (sieve arch)	ca	QC
	*Psidium cattleyanum* Sabine—MA094*	aracá	arco, flecha, bodoque - artesanato (crafts, bow, arrow, bodoque - like a slingshot)	ca	QC
Nyctaginaceae	*Guapira nitida* (Mart. ex J.A.Schmidt) Lundell—MA045*	carne-seca	fazer pião (make a spinning top, a wooden toy)	ca	QC
Poaceae	*Bambusa* sp.—SB027	bambú	colher, copo (spoon, cup)	ca	QC
	*Merostachys argyronema* Lindm.—CC032*	taquara, taquara-de-lixa	artesanato (handicrafts)	ca	QC
Primulaceae	*Myrsine coriacea* (Sw.) R.Br. ex Roem. & Schult.—THS052*	capororoca	artesanato (handicrafts)	ca	QC
Rubiaceae	*Borreria verticillata* (L.) G.Mey.—THS022*	vassorinha	vassoura (broom)	fo	QC
	*Rustia formosa* (Cham. & Schltdl.) Klotzsch—MAS042*	manduberana	artesanato (handicrafts)	ca	QC
Rutaceae	*Zanthoxylum rhoifolium* Lam.—THS028*	mamica-de-porca	pião (spinning top, a wooden toy)	ca	QC
Sapindaceae	*Cupania* cf. *oblongifolia* Mart.—MA064*	cubatam	artesanato (handicrafts)	ca	QC
Sapotaceae	*Ecclinusa ramiflora* Mart.—THS154*	guacá	artesanatos como canoinhas, remo (crafts such as canoes, rowing)	ca	QC
Solanaceae	*Solanum pseudoquina* A.St.-Hil.—MA134*	pilotera	artesanato (handicrafts)	ca	QC
		piloteira	fazer vassouras de enfeite (make decorative brooms)	ca	QF
Typhaceae	*Typha domingensis* Pers.—GDS011*	taboa	tapete (mat)	fo	QC
		taboa	tapetes, cestas, galinhas de enfeite (rugs, baskets, ornamental chickens)	pt	QF
**Category “Shipbuilding”**					
Annonaceae	*Annona dolabripetala* Raddi—THS034*	araticum	canoa (canoe)	ca	QC
	*Xylopia brasiliensis* Spreng.—MAS031*	canafista	canoa (canoe)	ca	QC
Apocynaceae	*Malouetia cestroides* (Nees ex Mart.) Müll. Arg.—MAS034*	guairana	canoa (canoe)	ca	QC
Araliaceae	*Schefflera* cf. *angustissima* (Marchal) Frodin—MA116*	imbirotó	canoa (canoe)	ca	QC
Bignoniaceae	*Handroanthus albus* (Cham.) Mattos—PBY090*	ipe-amarelo	canoa (canoe)	ca	QC
Boraginaceae	*Aniba* sp.—THS09	loro	barco e remo (boat and oar)	ca	QF
	*Cordia* sp.1—MAS085	louro	canoa (canoe)	ca	QC
	*Cordia* sp.2—THS080	louro-pardo	canoa (canoe)	ca	QC
	*Cordia* sp.3—THS081	louro-pardo	canoa (canoe)	ca	QC
Clusiaceae	*Clusia criuva* subsp. parviflora Vesque—THS120*	figueira-braçadeira	canoa (canoe)	ca	QC
Combretaceae	*Buchenavia kleinii* Exell—THS046*	angelim	canoa (canoe)	ca	QC
Euphorbiaceae	*Actinostemon verticillatus* (Klotzsch) Baill.—MAS043*	sucanga	canoa (canoe)	ca	QC
	*Mabea piriri* Aubl.—MA047*	cano-de-pito	canoa (canoe)	ca	QC
	*Tetrorchidium* sp.—THS062	bapeva	canoa (canoe)	ca	QC
Fabaceae	*Albizia pedicellaris* (DC.) L.Rico—PBY56*	timbuíba	barco e remo (boat and oar)	ca	QF
	cf. *Hymenolobium janeirense* Kuhlm.—MA060*	guacuí, guiti	canoa (canoe)	ca	QC
	*Schizolobium parahyba* (Vell.) S.F.Blake—GDS60*	bacaurubu or guapuruvu	canoa (canoe)	ca	QF
	*Hymenaea* cf. *altissima* Ducke—MAS009*	jatobá	canoa (canoe)	ca	QC
	*Inga* cf. *lenticellata* Benth.—MAS027*	ingá-ferro	canoa (canoe)	ca	QC
	*Pseudopiptadenia leptostachya* (Benth.) Rauschert—MA049*	cobi	canoa (canoe)	ca	QC
	*Tachigali paratyensis* (Vell.) H.C.Lima—MA129*	ingá-flecha	canoa (canoe)	ca	QC
	*Tachigali* sp.1—MA054	ingá-amarelo	canoa (canoe)	ca	QC
	*Tachigali* sp.2—MA055	ingá-amarelo	canoa (canoe)	ca	QC
	*Tachigali* sp.3—THS027	ingá-amarelo (fedido)	canoa (canoe)	ca	QC
Lacistemataceae	*Lacistema lucidum* Schnizl—MAS040*	tatuzinho	canoa (canoe)	ca	QC
Lauraceae	*Nectandra oppositifolia* Nees—MA103*	canela-do-mato	tábua, barco, esteio (board)	ca	QC
Lecythidaceae	*Cariniana estrellensis* (Raddi) Kuntze—MAS038*	jequitibá	canoa (canoe)	ca	QC
Meliaceae	*Cabralea canjerana* (Vell.) Mart.—MAS052*	ingá-cajarana	canoa (canoe)	ca	QC
	*Cedrela* cf. *odorata* L.—MA104*	cedro	canoa (canoe)	ca	QC
	*Cedrela fissilis* Vell.—MAS055*	cedro-rosa	canoa (canoe)	ca	QC
		cedro rosa	canoa e remo (canoe and paddle)	ca	QF
Moraceae	*Brosimum guianense* (Aubl.) Huber—MA058*	guaricica-da-vermelha	canoa (canoe)	ca	QC
	*Ficus adhatodifolia* Schott—GDS64*	figueira	canoa (canoe)	ca	QF
		figueira-branca	canoa (canoe)	ca	QC
	*Ficus gomelleira* Kunth & C.D.Bouché—MA135*	figueira-parda	canoa (canoe)	ca	QC
	*Sorocea* cf. *guilleminiana* Gaudich.—THS171*	espinheira-santa	canoa (canoe)	ca	QC
Myristicaceae	*Virola bicuhyba* (Schott ex Spreng.) Warb.—MA113*	bicuíba	canoa (canoe)	ca	QC
Myrtaceae	*Eugenia* cf. *multicostata* D.Legrand—THS032*	carambola-do-mato	canoa (canoe)	ca	QC
Phyllanthaceae	*Hyeronima alchorneoides* Allemão—MAS035*	aricurana	canoa (canoe)	ca	QC
Primulaceae	*Myrsine coriacea* (Sw.) R.Br. ex Roem. & Schult.—THS052*	capororoca	barco (boat)	ca	QC
Sapindaceae	*Cupania* cf. *oblongifolia* Mart.—MA064*	cubatam	canoa (canoe)	ca	QC
Sapotaceae	*Ecclinusa ramiflora* Mart.—THS154*	guacuá	canoa (canoe)	ca	QC
	*Micropholis crassipedicellata* (Mart. & Eichler) Pierre—MAS053*	bacubixaba	canoa (canoe)	ca	QC
	*Pouteria* sp.1—THS063	guacuauçu	canoa (canoe)	ca	QC
	*Pouteria* sp.2—THS064	guacuáuçu	canoa (canoe)	ca	QC
Urticaceae	*Cecropia glaziovii* Snethl.—THS178*	cobi (do branco)	canoa (canoe)	ca	QC
	*Pourouma guianensis* Aubl.—THS162*	baubu	canoa (canoe)	ca	QC
**Category “Technology”**					
Annonaceae	*Guatteria australis* A.St.-Hil.—PBY13*	astro-de-fisga	fisga para pescar peixe (bait to catch fish)	ca	QF
Araceae	*Philodendron eximium* Schott—GDS29*	imbé	cabos de rede (network cables)	li	QF
	*Philodendron martianum* Engl.—SB30*	banana-do-mato	crescer cabelo, deixar brilhante (grow hair, make it shiny)	fo	QF
	*Xanthosoma taioba E.G*.Gonç.—MA089*	taioba	Usado como veneno para matar a caça. Colocar junto com o chumbo, na espingarda (Used as a poison to kill game. Put along with the lead, in the shotgun)	tu	QC
Arecaceae	*Astrocaryum aculeatissimum* (Schott) Burret—GDS74*	brejaúba	móveis (furniture)	ca	QF
Asparagaceae	cf. *Furcraea foetida* (L.) Haw.—SB59	pita	a linhazinha do meio era usada para costurar (the middle thread was used for sewing)	fo	QF
Asteraceae	*Achyrocline* sp.1—MA041	marcela	fazer travesseiro (make pillow)	fl	QC
	*Achyrocline* sp.1—PBY32	macela	fazer travesseiro (make pillow)	pt	QF
	*Baccharis* sp. (Sect. Caulopterae DC.)–PBY74	carqueja	fazer espuma do sabão feito a partir de cinzas (make soap foam made from ash)	fo	QF
	*Cyrtocymura scorpioides* (Lam.) H.Rob.— MA082*	mata-pasto	abelhas são atraídas pelo néctar (bees are attracted to nectar)	fl	QC
Bixaceae	*Bixa orellana* L.—SB26*	urucum	fazer corante pra colocar na comida (make food coloring)	se	QF
Clusiaceae	*Clusia criuva* subsp. parviflora Vesque—THS120*	mangue	âncora para segurar cerco, usado como armadilha para pegar peixes (anchor to hold siege, used as a trap to catch fish)	ca	QC
Erythroxylaceae	*Erythroxylum pulchrum* A.St.-Hil.—PBY04*	guará-cipó	cabos de ferramenta (tool handles)	ca	QF
Euphorbiaceae	*Mabea piriri* Aubl.—MA047*	cano-de-pito	cabo de machado (ax handle)	ca	QC
Fabaceae	cf. *Pterocarpus rohrii* Vahl—PBY07*	guaricica or guaricica-amarela	cabo de ferramentas (tool handles)	ca	QF
	*Swartzia simplex* var. grandiflora (Raddi) R.S.Cowan—PBY08*	laranjeira-do-mato; canela-prego	cabo de machado, móveis (ax handle furniture)	ca	QF
		laranjeira-do-mato	essência, cheirosa (essence, fragrant)	fl	QC
	*Canavalia ensiformis* (L.) DC.—MA030	feijão-de-porco	adubo para a terra (compost for the land)	pt	QC
	*Tephrosia candida* (Roxb.) DC.—MA024	trifosa	adubo (compost)	ra	QC
Lacistemataceae	*Lacistema lucidum* Schnizl–PBY06*	burrachudo	cabos de ferramenta (tool handles)	ca	QF
Lamiaceae	*Aegiphila integrifolia* (Jacq.) Moldenke—MAS036*	cajuja, maria-mole	sabão de lavar roupa (washing soap)	fo	QC
Lauraceae	*Cryptocarya saligna* Mez—PBY24*	canela-sassafraize	móveis (furniture)	ca	QF
Malvaceae	*Gossypium* sp.—SB029	algodão	travesseiro (pillow)	fr	QC
	*Sida planicaulis* Cav.—PBY91*	vassoura-guanxuma	fazer sabão de cinzas (make soap from ash)	fo	QF
	*Sida rhombifolia* L.—GDS19*	vassoura-guanxuma	fazer sabão de cinzas (make soap from ash)	fo	QF
Marantaceae	*Ctenanthe lanceolata* Petersen—THS023*	caitê	usa a folha para assar peixe, fazer pamonha (uses the leaf to bake fish, make pamonha)	fo	QC
	*Goeppertia* sp.—GDS48	caité or assapeixe	para embrulhar pamonha, assar peixe ou fazer copo para tomar água (to wrap tamale, bake fish or make a glass to drink water)	fo	QF
Melastomataceae	*Huberia ovalifolia* DC—MA108*	tinteiro	tingir redes (dye nets)	cs	QF
		tinteiro	tinta com a casca, para tingir rede, roupa (paint with bark, to dye net, clothes)	ca	QC
	*Tibouchina pulchra* Cogn.—MA110*	quaresmeira	Fazer tinta com a casca (Make ink with the shell)	ca	QC
Meliaceae	*Guarea macrophylla* Vahl—MA96*	café-do-mato	cabo de ferramenta (tool handles)	ca	QC
Moraceae	*Brosimum guianense* (Aubl.) Huber—MA058*	guaricica-da-vermelha	cabo de machado (ax handle)	ca	QC
	*Ficus adhatodifolia* Schott—MA121*	figueira-branca	lavar roupas (washing clothes)	fo	QC
	*Morus nigra* L.—GDS068	amora	fazer seda (make silk)	fo	QC
	*Sorocea* cf. *guilleminiana* Gaudich.—PBY09*	garapinha	cabo de ferramentas (tool handles)	ca	QF
Myrtaceae	*Eugenia* cf. *stipitata* McVaugh—GDS73	araça-do-norte or de morcego	bodoque e puçá (bodoque - like a slingshot, and bow for fishing instrument frame)	ca	QF
	*Myrcia* sp.1—MA114	arco-de-peneira	cabo de ferramentas, como machado e foice; fazer peneira para pescar (tool handles such as ax and sickle; make a sieve for fishing)	ca	QC
	*Myrcia spectabilis* DC.—PBY05*	arco-de-peneira	cabos de ferramenta (tool handles)	ca	QF
	sp. 2—PBY12	muta	bodoque e arco de puça (bodoque - like a slingshot, and bow for fishing instrument frame)	ca	QF
Piperaceae	*Piper cernuum* Vell.–THS031*	papel-higiênico	papel higiênico (toilet paper)	fo	QC
	*Piper cf*. gaudichaudianum Kunth—MAS026*	jubrandi	shampoo (shampoo)	fo	QC
Poaceae	*Merostachys argyronema* Lindm.—CC032*	taquara-de-lixa	covo para pegar peixe no mar (cave to catch fish in the sea)	ca	QC
Primulaceae	*Myrsine coriacea* (Sw.) R.Br. ex Roem. & Schult.—THS052*	capororoca	Cobrir a terra com as folhas para aumentar a umidade (cover the soil with leaves to increase moisture)	fo	QC
Rubiaceae	*Faramea hymenocalyx* M.Gomes—PBY11*	catinga-de-porca	vara para pescar (fishing rod)	ca	QF
	*Rustia formosa* (Cham. & Schltdl.) Klotzsch—PBY25*	guacá	colher de pau e cabo de ferramentas (wooden spoon and tool handle)	ca	QF
	*Zanthoxylum rhoifolium* Lam.—GDS54*	mamica-de-porca or manataru	cabos de ferramenta (tool handles)	ca	QF
Sapindaceae	*Cupani*a cf. *oblongifolia* Mart.—MA064*	cubatam	cabo de machado (ax handle)	ca	QC
Sapotaceae	*Ecclinusa ramiflora* Mart.—THS154*	guacuá	usada para pegar passarinho (used to catch birds)	ex	QC
Solanaceae	*Solanum pseudoquina* A.St.-Hil.—MA134*	pilotera	essência para sabonete (essence for soap)	fl	QC
**Category “Ritual”**					
Meliaceae	*Cedrela fissilis* Vell.—SB34*	cedro-rosa	simpatias (sympathies)	cs	QF
Moraceae	*Ficus adhatodifolia* Schott—GDS64*	figueira	simpatias (sympathies)	pt	QF
**Category “Ornamental”**					
Marantaceae	*Marantaceae* sp.1—THS114	caitê	ornamental (ornamental)	pt	QC
Zingiberaceae	*Renealmia petasites* Gagnep.—THS030*	pacova	ornamental (ornamental)	pt	QC

**Notes:**

The same species can be present in more than one category simultaneously.

Websites for botanical databases: “Flora do Brasil 2020” (available at: https://floradobrasil.jbrj.gov.br/) and “The Plant List” (available at: http://www.theplantlist.org/), were consulted to obtain the accepted and updated botanical nomenclature. The former was also consulted to determine if particular species were native to the Atlantic Forest; these are highlighted with asterisks in Table 1.

Sixty-three botanical families were identified in the two communities, the most frequent being Fabaceae (24) and Myrtaceae (21). With 210 genera and 2,694 species, Fabaceae is considered to hold great ecological relevance by some authors and is the most significant family in Brazil (Cavalheiro, Peralta & Furlan, 2003; Forzza et al., 2010). Myrtaceae, on the other hand, is represented in the state of São Paulo by approximately 150 species (Wanderley et al., 2011) (Table 1).

The most frequently cited species were *Mabea piriri* Aubl (mentioned 11 times, nine in the QC and two in the QF), *Swartzia simplex* var. *grandiflora* (Raddi) R.S.Cowan (mentioned nine times, four in the QC and five in the QF), *Euterpe edulis* Mart. (mentioned nine times, five in the QC and four in the QF) and *Ecclinusa ramiflora* Mart (mentioned eight times, all of which were cited in the QC). Of the total species, 59.34%, indicated with the asterisk in Table 1, are native to the Atlantic Forest ecosystem, while the others are introduced, or have not been fully identified. This is of great relevance because it reflects a greater use of local native species by residents, which is not seen in most studies of ethnobotany, in which the greatest use by communities has often been found for exotic species (Pinto, Amorozo & Furlan, 2006; Begossi, Hanazaki & Tamashiro, 2002).

However, this finding demonstrates the diversity of species that can be found in the region, reflecting the great biodiversity of Brazil, which has an estimated flora of 41,000 species (Martinelli & Moraes, 2013). *Euterpe edulis* (palmiteiro) and *Mabea piriri* (canudo-de-pito) are frequent species in the areas of use of both the QC and the QF (Conde et al., 2020).

The following numbers of species per category were indicated in each quilombo: “ornamental” (two species QC and zero QF); “food/spices” (72 and 71); “technology” (25 and 24); “combustion” (18 and six); “ritual” (zero and two); “civil construction” (45 and 33); “shipbuilding” (42 and five) and “handicrafts” (33 and 14), see Fig. 2. While the number of coincident species cited in both quilombos were: “ornamental” (zero species); “food/spices” (23); “technology” (two), “combustion” (one), “ritual” (zero), “civil construction” (11), “shipbuilding” (two) and “handicrafts” (four), Fig. 2.

Figure 2 shows that in general, the categories containing the highest numbers of species indicated were “food/spices” (72 QC and 71 QF) and “civil construction” (45 QC and 33 QF), consistent with the findings of other investigations, that have also presented these categories showing greater numbers of species (Crepaldi & Peixoto, 2010; Miranda et al., 2011; Baptista et al., 2013). Many of the ethnobotanical uses related to the construction of houses are somewhat neglected, possibly due to the complexity of the use of timber resources and less sustainability involved, when compared to the non-timber resources, which are more often investigated (Kang et al., 2017). Traditional uses involving wood in these quilombos began before the Park was established in the region, when logging was not restricted.

The “food/spice” category featured the greatest number of species shared between quilombos (23 species). This can be attributed to an adaptation of the first residents who arrived at these places in the face of geographical isolation from the nearest urban centers. Since at the time there were no roads, they began to use the forest to meet their needs. This may also be a reflection of the fact that the cultivation of land is the basis of local subsistence, which remains to this day and reinforces the relationship of these communities with the environment in which they live.

**Figure 3:**
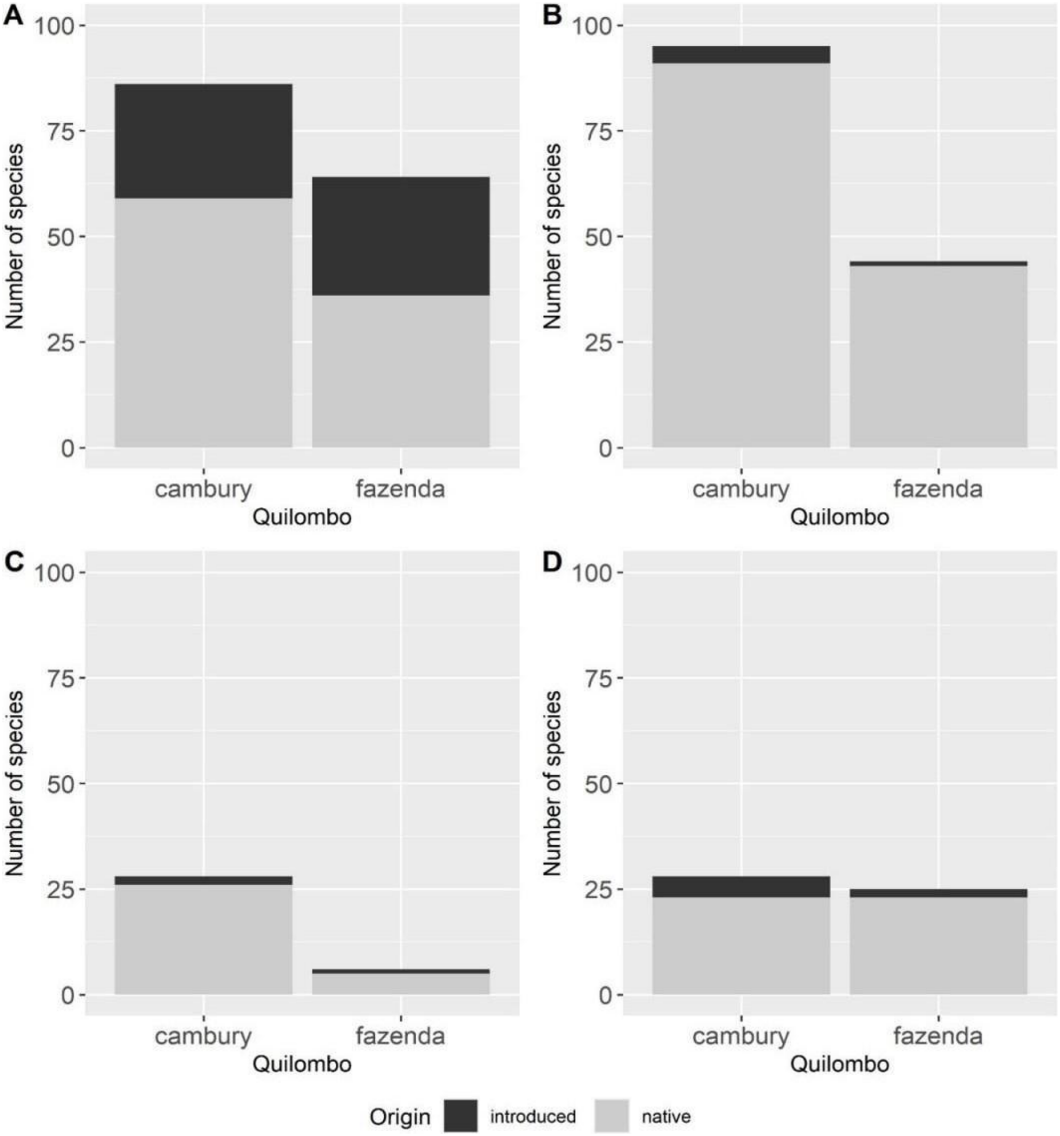
Comparison between the number of introduced *vs*. native species cited by local experts from Quilombo do Cambury and Quilombo da Fazenda, in the Serra do Mar State Park, Southeastern Brazil. Categories: “food/spices” (A), “fibers in technology” (B), “combustion” (C) and “civil construction” (D).

Most of the species known to experts from both quilombos for all categories of use are native; however when we compare the proportions of native and introduced species in both quilombos, we observe that there are no differences, considering all categories of use, including “food/spices” (χ^2^ = 1.91, *p* = 0.17), “fibers in technology” (χ^2^ = 0.0006, *p* = 0.93), “combustion” (χ^2^ = 7.09e−30, *p* = 1) and “civil construction” (χ^2^ = 2.70, *p* = 0.10) (Fig. 3).

**Figure 4:**
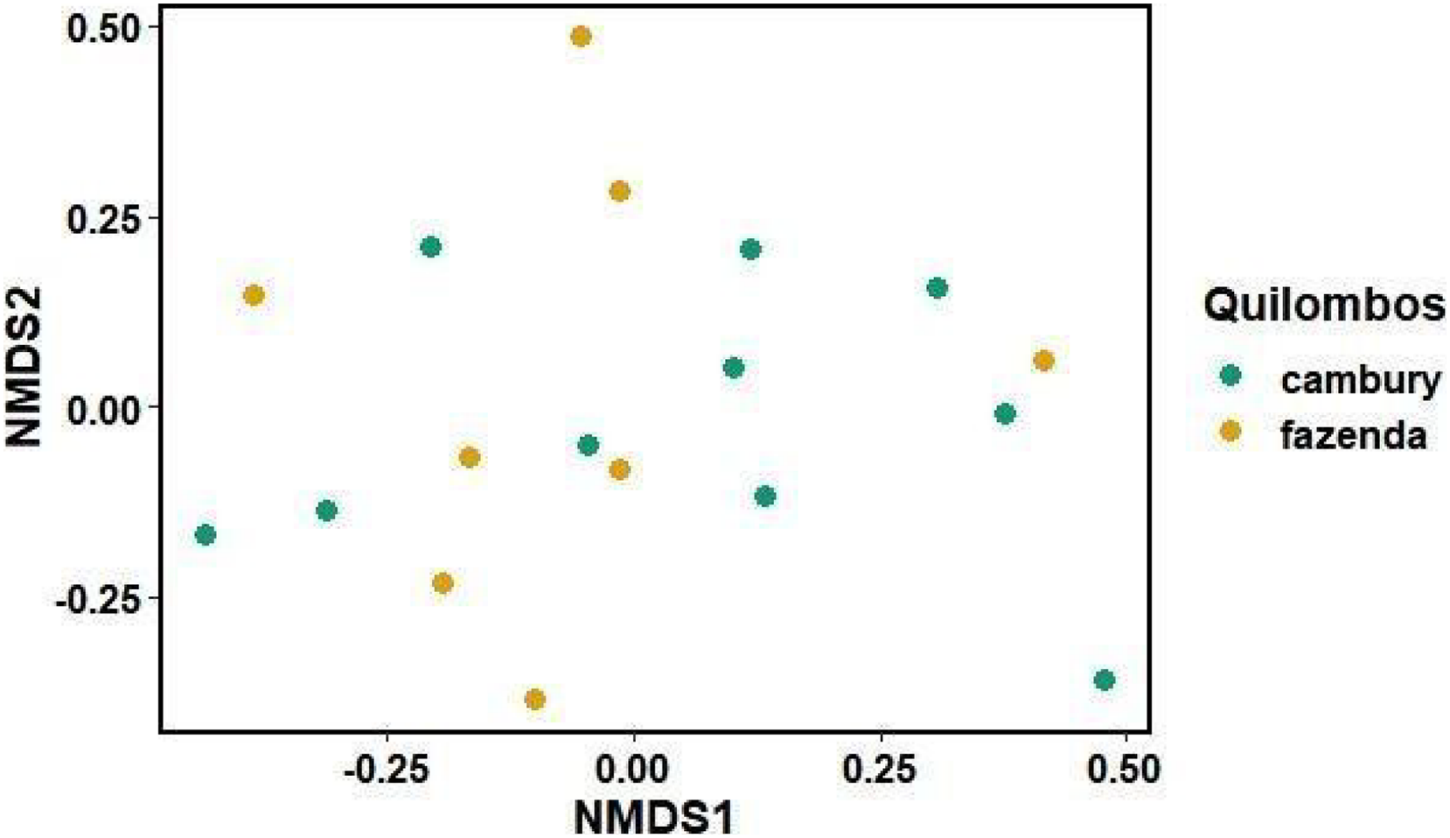
Non-metric multidimensional scaling graph (nMDS), comparing the dissimilarity of species (category “food/spices”) by local experts from Quilombo Cambury and Quilombo da Fazenda, in Serra do Mar State Park, Brazil. Stress = 0.1.

Different patterns were observed for each of the categories between quilombos in terms of species composition and beta diversity. For the category “food/spices”, there were no differences between quilombos in species composition (R^2^ = 0.06 F = 1.17 *p* = 0.12) or in beta diversity (F = 0.65 *p* = 0.44) (Fig. 4). Additionally, it is possible to verify that there was a marked disparity (observed by the distances between points in the figure) in the repertoire of species known among to local experts from both communities (Fig. 4). Some of the species cited by local experts of both quilombos included *Eryngium foetidum* L., *Coffea arabica* L., *Euterpe edulis* Mart., *Morus nigra* L. and *Myrciaria glazioviana* (Kiaersk.) G. M. Barroso ex Sobral (Fig. 4).

**Figure 5:**
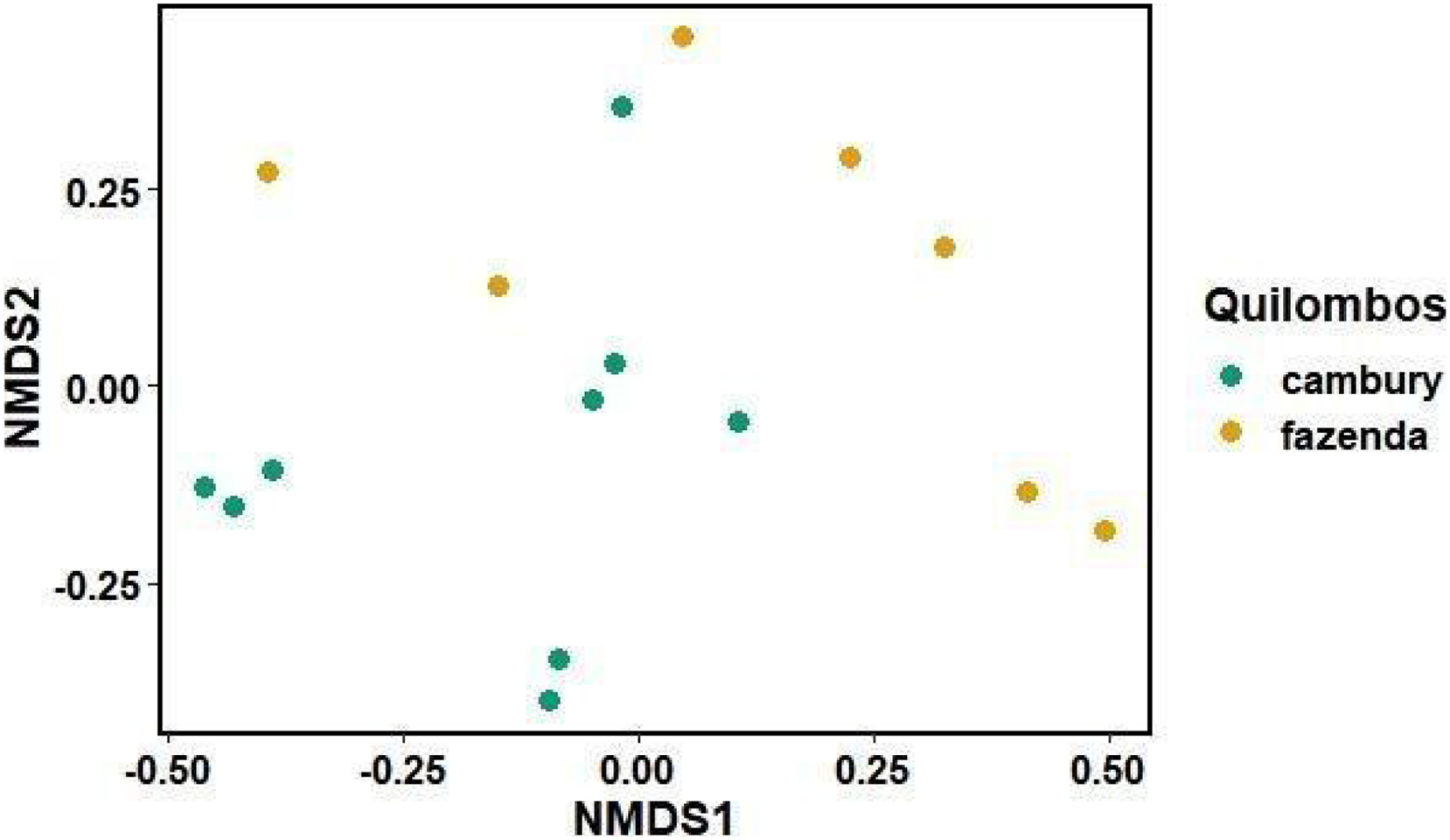
Non-metric multidimensional scaling graph (nMDS), comparing the dissimilarity of species known for “fibers in technology” (with handicrafts and shipbuilding) by local experts from Quilombo do Cambury and Quilombo da Fazenda, in Serra do Mar State Park, Br. Stress = 0.06.

For the category “fibers in technology”, it was observed that part of the variation in species composition was due to differences between quilombos (R^2^ = 0.09 F = 1.57 *p* = 0.002) (Fig. 5). Furthermore, for the “food/spices” use category, no differences were found between quilombos with respect to the beta diversity of known species (F = 0.24 *p* = 0.63) (Fig. 4). These findings indicate that there are species used for “fibers in technology” that are peculiar to each quilombo, probably because the vegetation found in each quilombo and/or the activities developed by its members are peculiar, since, while the QF is located in a “sertão” region with wetlands, the QC is on the edge near the beach, featuring more extensive areas of montane rainforest (type of vegetation absent in the QF). Thus, there are floristic differences between quilombos due to the phytophysiognomic differences observed in relief/soils and in distance from the ocean. Still, food plants are usually herbs and can be easily transported from one quilombo to the other through exchanges of knowledge between its residents. Perhaps this explains why in the category “food/spices” the plants used in both quilombos show no differences. The trees used as fibers do not have the same ease of cultivation and could explain the difference found between the quilombos for the category “fibers in technology”, as well as other categories composed mainly of trees and shrubs; such as: “civil construction”.

Additionally, considering that each point on the graph (Fig. 5) represents a local expert, there is a wide variation in knowledge among individuals within each community. This is illustrated by the presence of four canoe builders in the QC still working in that craft, while in the QF canoes are no longer made; that is, the knowledge in the QC is more diverse because it features a greater number of species and their citations. Some of the species cited for “fibers in technology” in both quilombos include *Astrocaryum aculeatissimum* (Schott) Burret, *Cedrela fissilis* Vell. and *Ficus adhatodifolia* Schott, used to build furniture and boats and to wash clothes, respectively. *Cedrela fissilis* Vell. occurs in almost all Brazilian territory, and its wood has also been extensively explored over the years due to its high commercial value, which is also observed in the communities; this contributes to the consequent decrease in the population of this species, leaving it at risk of extinction (Ruschel et al., 2003; Martinelli & Moraes, 2013; Flora do Brasil, 2020).

For the category of “civil construction”, there were differences in the composition of known species between local experts (R^2^ = 0.15 F = 1.25 *p* = 0.016). In addition, there were differences in the multivariate dispersion regarding the knowledge of quilombo local experts (F = 9.38 *p* = 0.026). Thus, there are species used for construction that are typical of each quilombo. In addition, the knowledge of the individuals in the QF was more homogeneous, while there was a marked variation in knowledge among the individuals in the QC. Some of the shared species used for construction, mentioned in both quilombos, included *Aniba* sp., *Hyeronima alchorneoides* Allemão and *Mabea piriri* Aubl.

**Figure 6:**
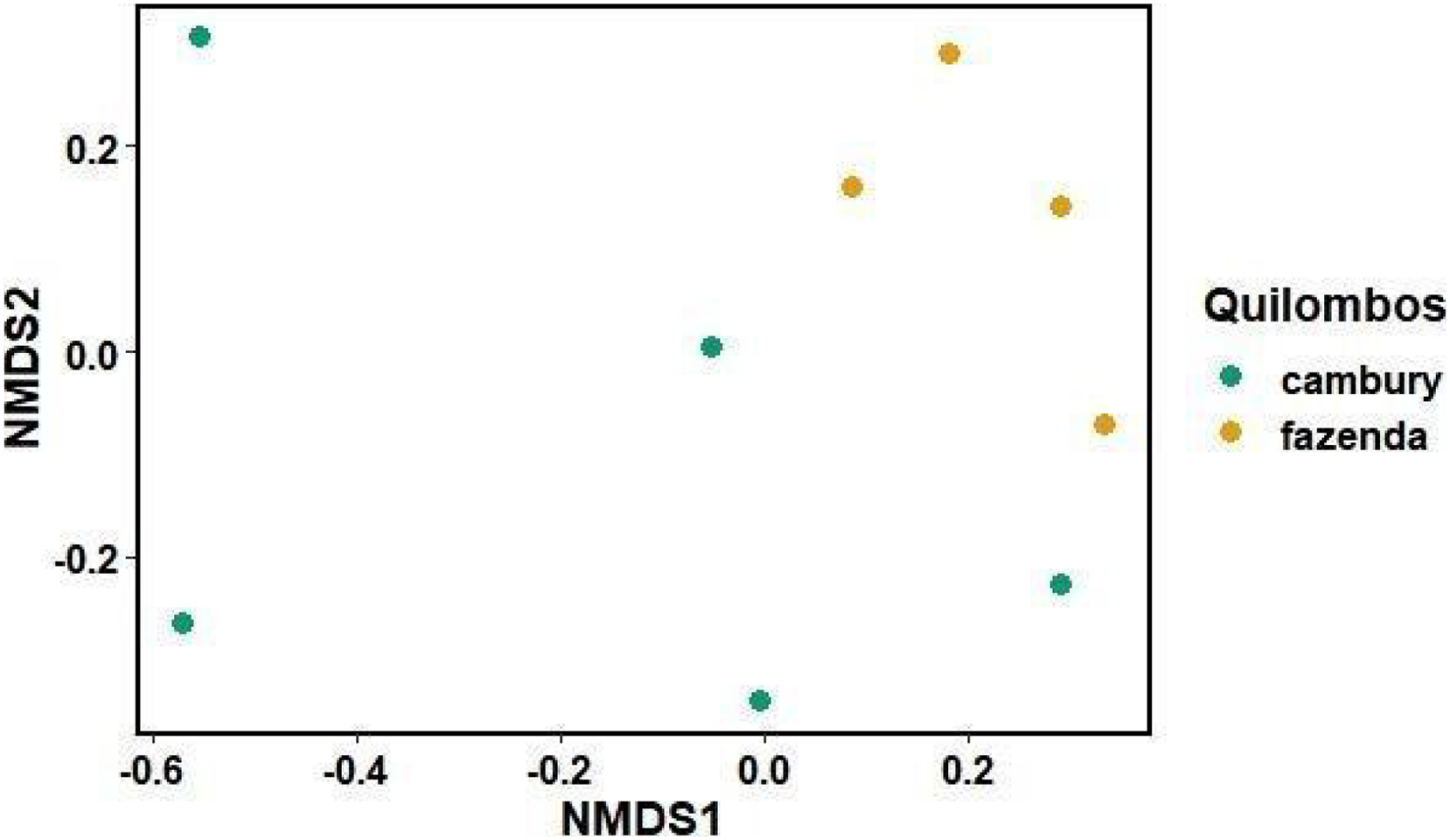
Non-metric multidimensional scaling graph (nMDS), comparing the dissimilarity of species known for civil construction by local experts from Quilombo Cambury and Quilombo da Fazenda, in the Serra do Mar State Park, Brazil. Stress = 0.07.

Although we have not compared the repertoire of plants known for “combustion” between the quilombos, only two species were mentioned in both, *Pera glabrata* (Schott) Poepp. ex Baill. and *Swartzia simplex* var. *grandiflora* (Raddi) R.S.Cowan. The low value of coincidences in this category may be associated with the reduced number of trees indicated by the members of each quilombo: 6 in QF and 18 in QC. Still, the low number of plants indicated in this category is probably related to the restriction imposed by the Park on residents regarding the extraction of trees (Fig. 6).

**Figure 7:**
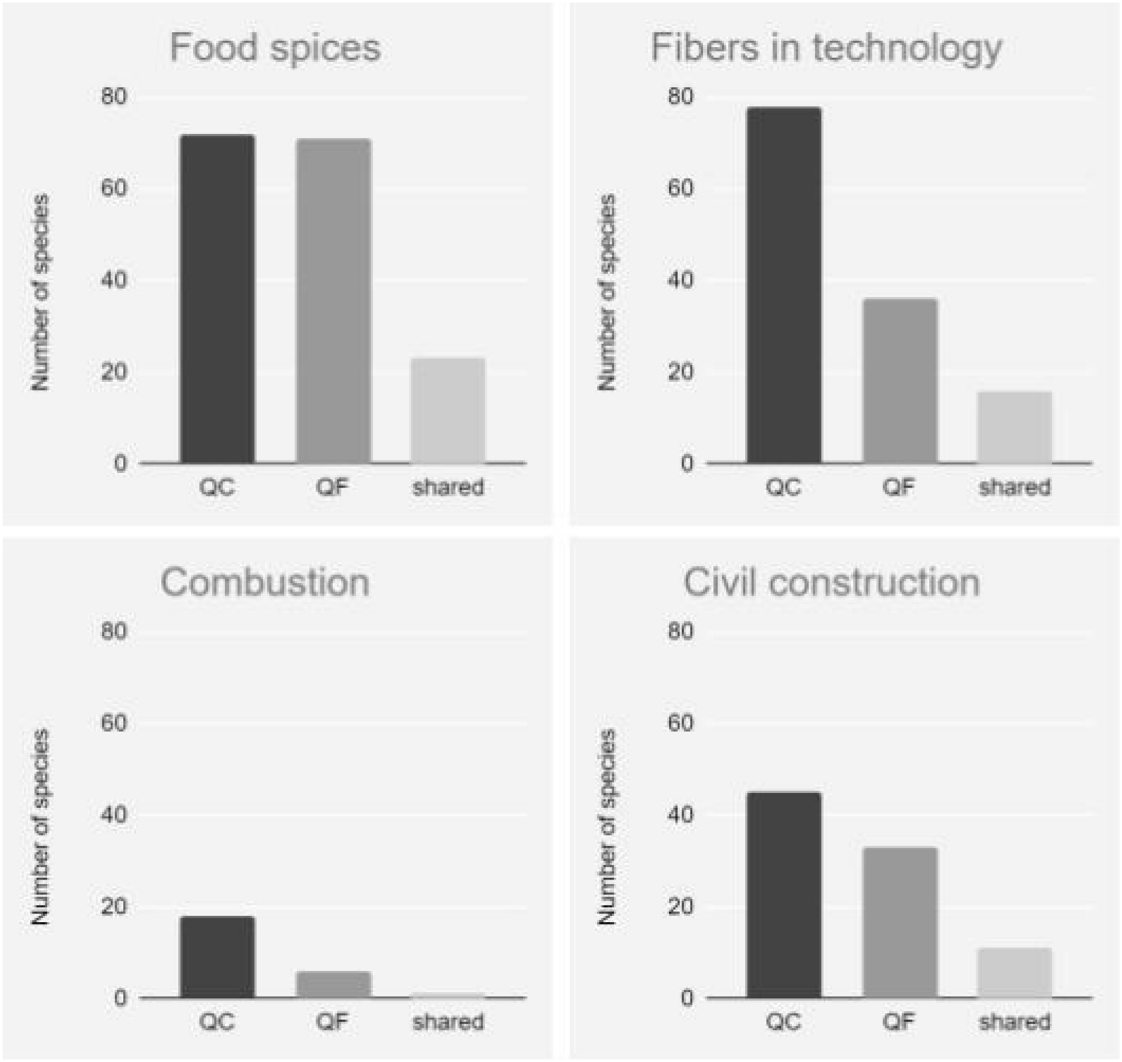
Comparison between the number of exclusive and shared plant species between Quilombo Cambury and Quilombo da Fazenda, in the Serra do Mar State Park, Brazil. Usage categories: “food/spices”, “fibers in technology”, “combustion” and “civil construction”.

In quantitative terms, there are few species that are known in both quilombos, with the following proportions for each of the categories of use: “food/spices” (19,16%), “technology” (16,27%), “combustion” (4,34%) and “civil construction” (16,41%) (Fig. 7).

The findings in the present study demonstrate that there are no differences in the proportion of native species between traditional communities with different social and environmental backgrounds. With respect the categories “fibers in technology”, “civil construction” and “combustion”, the ethnobotanical literature demonstrates that the selection of introduced species may be associated with the decline of native tree populations that have high quality wood (Nascimento et al., 2009; Medeiros et al., 2011; De Santana, Voeks & Funch, 2016). For example, Medeiros et al. (2011) observed a high frequency of chairs made from wood of introduced species (including coconut and jackfruit) in rural communities that reside in a highly fragmented Atlantic Forest landscape. In a community in the Brazilian semiarid region, Nascimento et al. (2009) noted that mesquite (*Prosopis juliflora* (Sw.) DC.) is one of the most used species for building fences. In general terms, these timber uses demand a large biomass of plant resources, so that the choice of species introduced for these uses can be affected by ecological characteristics of the species, such as high local availability, rapid growth and high capacity for resprouting.

The “food/spices” category, in turn, may have completely different entry dynamics for introduced species. It is possible that the Italian influence on the formation of the QF did not determine a greater proportion of introduced species than in the QC, since the entry of most cosmopolitan food species in Brazil occurred a long time ago. Nevertheless, in the 16th century, the first Brazilian settlers made great efforts to introduce and acclimatize food plants known from the Old World (De Santana, Voeks & Funch, 2016). The colonists were quickly successful in acclimating many species. In 1587, the chronicler Gabriel Soares de Sousa described a wide variety of European and Asian food plants already cultivated in the state of Bahia, such as orange, lemon, fig, palms, pomegranate, coconut and vines (Sousa, 1587). In his study of the history of food in Brazil, Cascudo (2014) argues that many of Brazil’s native fruits were little appreciated by Portuguese colonists (although some were consumed in the form of sweets), whereas fruits already known to Europeans (before the conquest of South American) were considered sophisticated and were frequent in the colonists’ meals.

This small difference observed may also be because the Park’s (PESM) zoning prohibits the use of exotic species in its territory, since they can disturb the Park’s ecosystem, so the management of these species is considered essential for strategies of local biodiversity conservation (São Paulo, 2006).

Regarding the differences in the composition of known species in the QF and QC, an initial observation is the high discrepancy in knowledge of among individuals within each community. These findings are probably a consequence of the intentional selection of respondents. Each of the local experts was recognized as having extensive local knowledge in different fields of knowledge, such as handicrafts or shipbuilding. Thus, this difference is a consequence of the degree of specialization of individuals in different domains.

Although members of both communities show concerns about the loss of traditional knowledge, the intentional sampling used in this study does not allow us to verify if this is happening. Despite this, the recognition of several individuals as local experts indicates that people can share notions about who is a good cultural model for learning (Henrich & Broesch, 2011). Therefore, in the scenario studied, these local experts can contribute to the restoration of practices associated with traditional knowledge.

Despite these discrepancies in knowledge among local experts, we find differences in the composition of species used for “fibers in technology” and for “construction” between communities. The selection and differential use of species for timber purposes is usually influenced by the local availability of the species (for example, the density or basal area of the species in the forest fragments near the communities), since they are uses which less restrictive selection criteria (compared to food and medicinal uses) and require a large amount of plant biomass (Gonçalves, Albuquerque & Medeiros, 2016). In addition, the greater beta diversity found for knowledge about construction in the QC than in the QF may also be a consequence of environmental factors. In environments with greater availability of forest resources, people can adopt more selective collection patterns guided by preferences (Top et al., 2004). Thus, if individuals have different preference criteria for selecting species, they may be familiar with different species. Therefore, differences in knowledge among communities can be a consequence of differences in the structure of forest communities adjacent to human settlements, what answers our hypothesis.

These differences in the structure and floristic composition between the QF and QC vegetation were demonstrated by the quantitative (phytosociological) studies carried out in sections of the forest that are frequented by quilombolas for the collection of native species for different uses; these areas were called “areas of use” (Conde et al., 2020). With equal sampling areas and inclusion criteria, that is, 0.1 ha and DBH (trunk diameter at 1.30 m of soil) equal to or greater than 4.8 cm, the results point to differences that corroborate the differences found in the analyzes carried out in this study, that is, in the QC, the area of use is richer in species, 78 species, compared to the QF, where 64 species were registered. The number of individuals sampled in each area of use reinforces the difference in the structure of the forest; in QC, the vegetation is made up of more numerous and large trees medium, while in the QF the forest is made up of larger individuals, comparatively. A total of 214 individuals were sampled in the QC and 158 individuals in the QF (Conde et al., 2020). These differences in the structure and floristic composition of forests are explained for the history of differentiated use in the areas (type, time and intensity of exploration) and for the very biodiversity present in each area, provided by the climatic and soil conditions, peculiar to each environment. The differences in the knowledge of the two quilombola communities may reflect the availability of resources (number and quantity of species and size of trees) found in the areas of use.

Previoulsy we present both challenges and opportunities of using participatory methods in ethnobotanical studies (Rodrigues et al., 2020), and at the same time we showed that it is possible to train community members who wish to document their knowledge to support the process of ensuring that local knowledge is highly regarded. It was also observed that despite both communities have a similar ancestry (quilombolas), inhabit the same biome (10 km distance only between them), and share knowledge about the use of plants among them; some of the plant species are used only by one of the quilombos (see Fig. 7).

Nowadays the residents of these two communities know each other and exchange knowledge with each other, unlike what happened years ago. Contact between these residents was facilitated with the construction of roads close to them. Today, this communication occurs not only between members of these two communities, but also through researchers and tourists visiting the sites.

In the present study, we observed that the wealth of data collected, as well as their analyzes were facilitated by the great involvement of community representatives in this process: especially in the selection of respondents who in fact accumulate a great deal of knowledge about certain uses (“handicrafts”, “civil construction”, and so on), also by the local researchers pointing out data analysis strategies during the workshops; and finally for contributing by pointing out the particular areas and phytophysiognomies involved in the areas of plant collection of their day-to-day active participation of community representatives in this project.

## Conclusion

Even with some limitations, the innovative methodology of participatory ethnobotany, defined in the present work, contributed to the empowerment of community members with regard to the use of their available resources in the environment in which they live, while retaining the intellectual property rights over their own knowledge.

The large number of plant species cited provided us with greater current knowledge of the local biodiversity, reflecting the history of formation of each of the quilombos, their geographic locations, and all the relationships that their residents have developed with the resources available in the environment over the year.

This research favors an approximation of popular wisdom to scientific knowledge, allowing a better analysis of the use of local plant resources, which are important for the sustainable use of natural resources, promoting conservation and local development.

Most of the species known to experts of both quilombos, and for all categories of use, are native to Atlantic forest, and no significant differences were observed in the proportion of native species *vs*. introduced among quilombos for any of the categories of use studied. Still, these species present disparity in the patterns of composition between communities for the categories of use of “technology” and “civil construction”; and the QC has a greater number of species in these two categories. In addition, there was a variation of knowledge among experts within each community, reinforcing the influence of different geographic environments and different phytophysiognomies on the use of plants by different communities; and at the same time justifying our hypothesis. Thus, what our manuscript wants to show is that although the human groups in focus here have a similar origin and reside in an area of Atlantic forest, the phytophysiognomies found in each of the two areas can be determinant to define different knowledge.

## Supplemental Information

10.7717/peerj.16231/supp-1 Supplemental Information 1 Difference between the communities of the two quilombos raw data.

## Additional Information and Declarations

### Field Study Permissions

The following information was supplied relating to field study approvals (*i.e*., approving body and any reference numbers): For this research to be carried out, the following legal approvals were obtained: (1) To access the Serra do Mar State Park area (COTEC n° 260108–009.510/2015); (2) to collect plants and access the Serra da Bocaina National Park (SIBIO n° 51199-1/2015 and n° 51199-2/2015).

### Data Availability

The following information was supplied regarding data availability: The raw data is available in the Supplemental File.

## References

[ref-1] Albuquerque UP, De Medeiros PM, De Almeida ALS, Monteiro JM, de Freitas Lins Neto EM, de Melo JG, Dos Santos JP (2007). Medicinal plants of the caatinga (semi-arid) vegetation of NE Brazil: a quantitative approach. Journal of Ethnopharmacology.

[ref-2] Alexiades MN (1996). Selected guidelines for ethnobotanical research: a field manual.

[ref-3] Amorozo MCDM (2002). Use and diversity of medicinal plants in Santo Antonio do Leverger, MT, Brazil. Acta Botanica Brasilica.

[ref-4] Anderson MJ (2006). Distance-based tests for homogeneity of multivariate dispersions. Biometrics.

[ref-5] Baptista MM, Ramos MA, de Albuquerque UP, Coelho-de-Souza G, Ritter MR (2013). Traditional botanical knowledge of artisanal fishers in southern Brazil. Journal of Ethnobiology and Ethnomedicine.

[ref-6] Begossi A (1996). Use of ecological methods in ethnobotany: diversity indices. Economic Botany.

[ref-7] Begossi A, Hanazaki N, Tamashiro JY (2002). Medicinal plants in the Atlantic Forest (Brazil): knowledge, use and conservation. Human Ecology.

[ref-8] Bernard HR (1988). Research methods in cultural anthropology.

[ref-9] Brasil Constituição (2003). Decreto n° 4.887, de 20 de novembro de 2003. Diário Oficial da União, Brasília.

[ref-10] Canales M, Hernández T, Caballero J, Romo de Vivar A, Durán A, Lira R (2006). Análisis Cuantitativo del conocimiento tradicional de las plantas medicinales en San Rafael, Coxcatlán, Valle de Tehuacan-Cuicatlán, Puebla, México. Acta Botánica Mexicana.

[ref-11] Cascudo LC (2014). História da alimentação no Brasil.

[ref-12] Cavalheiro L, Peralta DF, Furlan A (2003). FIórula fanerogamica da planicie Iitoranea de Picinguaba, Ubatuba, SP, Brasi. HoehneaI.

[ref-13] Conde BE, Aragaki S, Ticktin T, Surerus Fonseca A, Yazbek PB, Sauini T, Rodrigues E (2020). Evaluation of conservation status of plants in Brazil’s Atlantic forest: an ethnoecological approach with Quilombola communities in Serra do Mar State Park. PLOS ONE.

[ref-15] Crepaldi MOS, Peixoto AL (2010). Use and knowledge of plants by “Quilombolas” as subsidies for conservation efforts in an area of Atlantic forest in Espírito Santo State, Brazil. Biodiversity and Conservation.

[ref-16] Cunha FC, Soares MGM, Fraxe TJP (2011). O Etnoconhecimento dos Pescadores sobre a Reprodução do Tucunaré (Cichla spp.) no Lago Grande de Manacapuru, AM. Saber do Norte.

[ref-17] De Almeida CDFCBR, Ramos MA, Silva RRV, de Melo JG, Medeiros MFT, Araújo TADS, de Albuquerque UP (2012). Intracultural variation in the knowledge of medicinal plants in an urban-rural community in the Atlantic Forest from Northeastern Brazil. Evidence-Based Complementary and Alternative Medicine.

[ref-18] De Santana BF, Voeks RA, Funch LS (2016). Ethnomedicinal survey of a maroon community in Brazil’s Atlantic tropical forest. Journal of Ethnopharmacology.

[ref-19] Ericson J (2006). A participatory approach to conservation in the Calak-mul Biosphere Reserve, Campeche, Mexico. Landscape and Urban Planning.

[ref-20] Etkin NL, Ticktin T (2005). Integrating ethnographic and ecological perspectives for ethnopharmacology field research. https://books.google.com.br/books?hl=pt-BR&lr=&id=4tjCDAAAQBAJ&oi=fnd&pg=PA96&dq=Etkin+NL,+Ticktin+T.+2005.+Integrating+ethnographic+and+ecological+perspectives+for+ethnopharmacology+field+research.+1.&ots=lWZvBoT8o7&sig=BUFnDjfl6dsj3flaTk8PYKQlokk#v=onepage&q&f=false.

[ref-21] Flora do Brasil (2020). em construção. Jardim Botânico do Rio de Janeiro. http://floradobrasil.jbrj.gov.br/.

[ref-22] Forzza RC, Leitman PM, Costa A, Carvalho JAAD, Peixoto AL, Walter BMT, Souza VC (2010). Catálogo de plantas e fungos do Brasil. Jardim Botânico do Rio de Janeiro.

[ref-23] FUNDART (2014). Quilombos. Prefeitura municipal de Ubatuba. https://fundart.com.br/tradicao/comunidades/quilombos/.

[ref-24] Fundação Cultural Palmares (2015). Quilombos. http://www.palmares.gov.br/.

[ref-25] Galleano G (2016). Forest use at the pacific coast quantitative approach. Economic Botany—The New York Botanical Garden Press, U.S.A.

[ref-26] Garibay-Orijel R, Caballero J, Estrada-Torres A, Cifuentes J (2007). Understanding cultural significance, the edible mushrooms case. Journal of Ethnobiology and Ethnomedicine.

[ref-27] Gilmore MP, Young JC (2012). The use of participatory mapping in ethnobiological research, biocultural conservation, and community empowerment: a case study from the Peruvian Amazon. Journal of Ethnobiology.

[ref-28] Goebel A (1998). Process, perception and power: notes from participatory research in a Zimbabwean resettlement area. Development and Change—The Hague then London.

[ref-29] Gonçalves PH, Albuquerque UP, Medeiros PM (2016). The most commonly available woody plant species are the most useful for human populations: a meta-analysis. Ecological Applications.

[ref-30] Grasser S, Schunko C, Vogl CR (2016). Children as ethnobotanists: methods and local impact of a participatory research project with children on wild plant gathering in the Grosses Walsertal Biosphere Reserve, Austria. Journal of Ethnobiology and Ethnomedicine.

[ref-31] Hanazaki N, Tamashiro JY, Leitão-Filho HF, Begossi A (2000). Diversity of plant uses in two Caiçara communities from the Atlantic Forest coast, Brazil. Biodiversity & Conservation.

[ref-32] Heinrich M, Williamson EM, Gibbons S, Barnes J, Prieto-Garcia J (2004). Fundamentals of pharmacognosy and phytotherapy.

[ref-33] Henrich J, Broesch J (2011). On the nature of cultural transmission networks: evidence from Fijian villages for adaptive learning biases. Philosophical Transactions of the Royal Society B: Biological Sciences.

[ref-34] Hitziger M, Heinrich M, Edwards P, Poll E, Lopez M, Krutli P (2016). Maya phytomedicine in Guatemala, can cooperative research change e thnopharmacological paradigms?. Journal of Ethnopharmacology.

[ref-35] Instituto Brasileiro de Geografia e Estatística (IBGE) (2012). Manual técnico da Vegetação Brasileira. Rio de Janeiro, RJ. https://biblioteca.ibge.gov.br/index.php/biblioteca-catalogo?view=detalhes&id=263011.

[ref-36] Instituto Brasileiro de Geografia e Estatística (IBGE) (2018). Biomass. https://www.ibge.gov.br/.

[ref-37] Instituto Chico Mendes de Conservação da Biodiversidade (ICMBio), Brasil (2018). Parque nacional serra da bocaina. https://www.icmbio.gov.br/parnaserradabocaina/.

[ref-38] Inventário Florestal do Estado de São Paulo (2020). Mapeamento da cobertura vegetal nativa. instituto florestal. São Paulo, SP. https://datageo.ambiente.sp.gov.br/.

[ref-39] ITESP (2002). Fundação Instituto Terras de São Paulo. Relatório técnico-científico sobre os remanescentes da comunidade de Quilombo de Camburi Ubatuba-SP. http://www.itesp.sp.gov.br/br/info/acoes/rtc/RTC_Cambury.pdf.

[ref-40] ITESP (2018). Fundação Instituto Terras de São Paulo. Assistência a Quilombos, Quilombos de São Paulo. http://www.itesp.sp.gov.br/br/info/acoes/assitencia_quilombos.aspx.

[ref-41] Johnson N, Lilja N, Ashby JA, Garcia JA (2004). The practice of participatory research and gender analysis in natural resource management. Natural Resources Forum. UK-USA.

[ref-42] Kang J, Kang Y, Feng J, Liu M, Ji X, Li D, Stawarczyk K, Łuczaj Ł (2017). Plants as highly diverse sources of construction wood, handicrafts and fibre in the Heihe valley (Qinling Mountains, Shaanxi, China): the importance of minor forest products. Journal of Ethnobiology and Ethnomedicine.

[ref-43] Kassambara A (2020). ggpubr: ggplot2 based publication ready plots. https://CRAN.R-project.org/package=ggpubr.

[ref-44] Martin GJ (2004). Ethnobotany: a methods manual.

[ref-45] Martinelli G, Moraes MA (2013). Livro vermelho da flora do Brasil.

[ref-46] Medeiros PM, Almeida ALS, Silva TC, Albuquerque UP (2011). Pressure indicators of wood resource use in an Atlantic Forest Area, Northeastern Brazil. Environmental Management.

[ref-47] Miranda TM, Hanazaki N, Govone JS, Alves DMM (2011). Existe utilização efetiva dos recursos vegetais conhecidos em comunidades caiçaras da Ilha do Cardoso, estado de São Paulo, Brasil. Rodriguésia.

[ref-48] Mosse D, Cooke B, Kothari U (2001). People’s knowledge, participation and patronage: operations and representations in rural development. Participation: The New Tyranny?.

[ref-49] Nascimento VT, Sousa LG, Alves AGC, Araújo EL, Albuquerque UP (2009). Rural fences in agricultural landscapes and their conservation role in an area of caatinga (dryland vegetation) in Northeast Brazil. Environment, Development and Sustainability.

[ref-50] Oksanen J, Blanchet G, Friendly M, Kindt R, Legendre P, McGlinn D, Minchin PR, O’Hara RB, Simpson GL, Solymos P, Stevens MHH, Szoecs E, Wagner H (2019). Vegan: community ecology package. R Package Version. 2:5–6. https://CRAN.R-project.org/package=vegan.

[ref-51] PESM (2018). Parque Estadual Serra do Mar. https://www.saopaulo.sp.gov.br/conhecasp/parques-e-reservas-naturais/parque-estadual-serra-do-mar/.

[ref-52] Pinto E, Amorozo MCM, Furlan A (2006). Conhecimento popular sobre plantas medicinais em comunidades rurais de mata atlântica-Itacaré, BA, Brasil. Acta Botanica Brasilica.

[ref-53] Prance GT, Balée W, Boom BM (1987). Quantitative ethnobotany and the Case for Conservation in Amazonia. Conservation Biology.

[ref-54] Rodrigues E, Cassas F, Conde BE, Da Cruz C, Barretto EHP, Dos Santos G, Figueira GM, Passero LFD, Dos Santos MA, Matta P, Yazbek P, Garcia JF, Braga S, Aragaki S, Honda S, Sauini T, Fonseca-Kruel VS (2020). Participatory ethnobotany and conservation: a methodological case study conducted with quilombola communities in Brazil’s Atlantic Forest. Journal of Ethnobiology and Ethnomedicine.

[ref-71] R Core Team (2020). R: a language and environment for statistical computing.

[ref-55] Ruschel AR, Nodari ES, Guerra MP, Nodari RO (2003). Evolução do uso e valorização das espécies madeiráveis da Floresta Estacional Decidual do Alto-Uruguai, SC. Ciência Florestal.

[ref-60] São Paulo (2006). Plano de Manejo do Parque Estadual Serra do Mar. Instituto Florestal e Secretaria do Meio Ambiente. http://www.mma.gov.br/areas-protegidas/unidades-de-conservacao/plano-demanejo.

[ref-56] Sauini T, Stern da Fonseca-Kruel V, Baptistela PY, Matta P, Cassas F, Da Cruz C, Barretto EHP, Dos Santos MA, Gomes MA, Garcia RJF, Sumiko H, Passero LFD, Conde BE, Rodrigues E (2020). Participatory methods on the recording of traditional knowledge about medicinal plants in Atlantic forest, Ubatuba, São Paulo, Brazil. PLOS ONE.

[ref-57] Setti K (1985). Ubatuba nos cantos das praias: estudo do caiçara paulista e de sua produção musical.

[ref-58] Sieber SS, da Silva TC, de Oliveira Campos LZ, Zank S, Albuquerque UP (2014). Participatory methods in ethnobiological and ethnoecological research. Methods and Techniques in Ethnobiology and Ethnoecology.

[ref-59] Sousa GS (1587). Tratado descritivo do Brasil. http://www.dominiopublico.gov.br/pesquisa/DetalheObraForm.do?select_action=&co_obra=38095.

[ref-61] Top N, Mizoue N, Kai S, Nakao T (2004). Variation in woodfuel consumption patterns in response to forest availability in Kampong Thom Province, Cambodia Neth. Biomass and Bioenergy.

[ref-14] Wanderley MDGL, Shepherd GJ, Martins SE, Estrada TEMD, Romanini RP, Koch I, Esteves GL (2011). Checklist das Spermatophyta do Estado de São Paulo, Brasil. Biota Neotropica.

[ref-62] Wickham H (2016). ggplot2: elegant graphics for data analysis.

[ref-63] Yazbek PB, Matta P, Passero LF, Dos Santos G, Braga S, Assunção L, Sauini T, Cassas F, Garcia RJF, Honda S, Barreto EHP, Rodrigues E (2019). Plants utilized as medicines by residents of Quilombo da Fazenda, Núcleo Picinguaba, Ubatuba, São Paulo, Brazil: a participatory survey. Journal of Ethnopharmacology.

